# Polarization-sensitive neuromorphic vision sensing enabled by pristine black arsenic-phosphorus

**DOI:** 10.1038/s41377-025-02125-0

**Published:** 2026-02-02

**Authors:** Shi Zhang, Shuguang Zhu, Shijian Tian, Libo Zhang, Cheng Chen, Kening Xiao, Wenqi Mo, Shicong Hou, Yunduo Zhang, Yuanfeng Wen, Yiran Tan, Kaixuan Zhang, Jiayue Han, Changlong Liu, Jiale He, Weiwei Tang, Jun Wang, Guanhai Li, Kai Zhang, Lin Wang, Xiaoshuang Chen

**Affiliations:** 1 College of Physics and Optoelectronic Engineering, Hangzhou Institute for Advanced Study, University of Chinese Academy of Sciences, No. 1, Sub-Lane Xiangshan, Xihu District, Hangzhou, 310024 China; 2 State Key Laboratory of Infrared Physics, Shanghai Institute of Technical Physics, Chinese Academy of Sciences, 500 Yu-Tian Road, Shanghai, 200083 China; 3 Key Laboratory of Semiconductor Display Materials and Chips & i-Lab, Suzhou Institute of Nano-Tech and Nano-Bionics (SINANO), Chinese Academy of Sciences, Suzhou, 215123 China; 4 Shanghai Key Lab of Modern Optical System, University of Shanghai for Science and Technology, 516 Jungong Road, Shanghai, 200093 China; 5 School of Optoelectronic Science and Engineering, University of Electronic Science and Technology of China, Chengdu, 610054 China; 6 Suzhou Laboratory, Suzhou, Jiangsu 215000 China

**Keywords:** Optical sensors, Micro-optics

## Abstract

Polarization-sensitive neuromorphic vision sensing excels in distinguishing light polarization states, offering intrinsic advantages in reducing glare and enhancing visual clarity in complex lighting environments, enabling advanced applications in autonomous driving, optical communication, and bioinspired imaging across the visible-to-infrared spectrum. Here, we present a polarization-sensitive neuromorphic phototransistor based on a high-quality, intrinsically anisotropic two-dimensional black arsenic-phosphorus nanosheet, which exhibits exceptional optoelectronic performance with a peak responsivity of 2.88 A W^-1^, a polarization ratio of 4.7 and a dynamic range of 40 dB within the near-infrared communication band. Through multidimensional input control, including polarization and gate voltage, the phototransistor successfully simulates synaptic behaviors analogous to human neural responses to visual stimuli, with paired-pulse facilitation values reaching 201%. Critically, the device demonstrates gate-tunable short-term plasticity, with optical persistence triggering stable long-term plasticity states that underpin memory consolidation. The neuromorphic properties enable the development of a hybrid optical-electronic neural network which achieves a classification accuracy of over 90% on the Fashion-MNIST dataset and a reconstruction accuracy of 71.38% using data from the Yale Face Database under 0º linear polarization. We demonstrate a polarization-resolved imaging approach utilizing the black arsenic-phosphorus phototransistor to reconstruct hidden targets with high fidelity through Stokes parameter extraction and degree of linear polarization mapping, revealing intricate polarization features invisible to conventional imaging systems. Our work establishes a foundational platform for high-performance neuromorphic vision systems with integrated polarization imaging, computation, and communication functionalities, addressing critical challenges in scalable brain-inspired optoelectronic technologies.

## Abstract (web summary)

An anisotropic b-As_0.2_P_0.8_ phototransistor enables polarization-tunable synaptic behaviors with 4.7 polarization ratio, achieving >90% classification accuracy and high-fidelity polarization imaging through Stokes parameter reconstruction and DoLP mapping.

## Graphical abstract

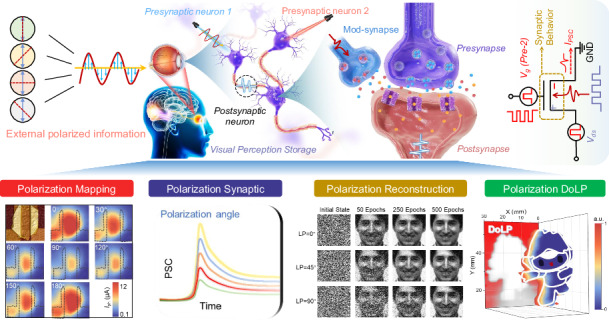

## Introduction

**Fig. 1:**
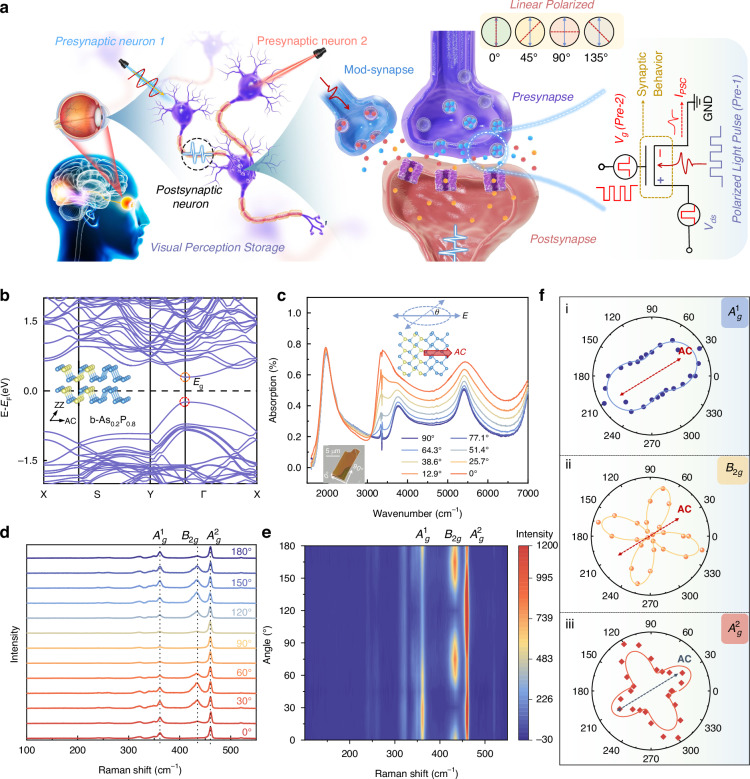
Artificial polarization synapse design and b-As_0.2_P_0.8_ crystal characteristics. **a** Schematic representation of a biological neural network with a synapse, alongside a simplified electrical circuit model for the optical synapse. **b** The band structure computed by the DFT method for b-As_0.2_P_0.8_ crystal. **c** Polarization-resolved absorption spectra of b-As_0.2_P_0.8_ crystal on the CaF_2_ substrate. **d** Angle-resolved polarized Raman spectra from 0° to 180° corresponding to three Raman peaks *A*_g_^1^, *B*_2g_, and *A*_g_^2^. **e** Contour mapping of angle-resolved polarized Raman spectra. **f** Polar plots for the Raman peak intensity at *A*_g_^1^, *B*_2g_, and *A*_g_^2^.with respect to *θ*, respectively

Bioinspired neuromorphic vision (NV) sensors draw inspiration from biological vision systems, such as the human eye, to mimic their ability to sense, process, and interpret visual information in real-time, embed computation and memory into the sensing process itself^1,2^, which not only reduces redundant data generation but also allows for localized, energy-efficient processing^3–5^, being particularly advantageous in scenarios requiring rapid decision-making, such as autonomous driving, robotic vision, and advanced surveillance systems^6,7^. Polarization-sensitive neuromorphic vision (P-NV) sensors play a crucial role in modern photonic technologies, as the polarization state of light encodes unique information beyond conventional parameters such as intensity, wavelength, or phase^8,9^. At their core, P-NV sensors emulate the function of polarization-sensitive photoreceptors found in biological systems, where incoming light is transformed into electrical signals and preprocessed as illustrated in Fig. 1a. However, unlike the human eye, which is largely insensitive to polarization except for subtle phenomena like Haidinger’s brush, these sensors actively detect and process polarization information, enabling advanced functionalities such as glare reduction, enhanced contrast, and the extraction of hidden details in complex environments. P-NV sensors represent a significant advancement by encoding additional information about the orientation of light waves, which are critical for applications ranging from autonomous navigation and optical communication to biomedical imaging and remote sensing^10–13^. However, bulk anisotropic crystals, such as birefringent materials, often exhibit limited integration potential due to their large dimensions and fixed optical properties, making them unsuitable for miniaturized, tunable devices. The traditional polarization-sensitive systems, reliant on bulky polarizers, filters, and external processing units, face significant challenges in miniaturization and integration, limiting their scalability for compact and portable devices^14–16^. While metamaterials achieve polarization sensitivity through tailored subwavelength geometries^17^, their performance is inherently constrained by lithographic resolution, narrow bandwidth and susceptibility to structural imperfections^18,19^.

Anisotropic two-dimensional (2D) materials offer intrinsic polarization sensitivity arising from their crystallographic symmetry, a property that fundamentally distinguishes them from traditional approaches reliant on artificially engineered structures^20,21^. Recent advances in 2D materials have transformed the landscape of photodetection pathways toward compact and efficient photodetection solutions^22–24^, offering solutions with atomic-scale thickness, enhanced light-matter interactions, and intrinsic in-plane anisotropy^25,26^. Emerging optoelectronic devices based on 2D materials have shown promise in mimicking synaptic behaviours through optical stimuli, enabling neuromorphic computations and image pre-processing. Kamaei et al. integrated a WSe_2_/SnSe_2_ heterostructure to develop a reconfigurable morphologic computing device, leveraging ferroelectric polarization to enable low-power, high-performance neuromorphic simulations^27^. By modulating the 2D channel threshold voltage through pulses, the device successfully replicated both long-term potentiation (LTP) and long-term depression (LTD). Based on this, Peng et al. employed a gate voltage-programmed gradient doping strategy to simulate synaptic functionality, demonstrating a broad range of synaptic plasticity, including homosynaptic, heterosynaptic, and spike-timing-dependent plasticity^28^. Meanwhile, Tsai et al. utilized a hBN/ReSe_2_/hBN heterojunction with optically induced trapping mechanisms to achieve non-volatile, tunable polarity regulation, replicating LTP and LTD behaviors, and integrating the device into an eight-layer convolutional neural network (CNN) for precise image recognition^29^. Additionally, Wang et al. proposed a 2D material-based infrared optoelectronic retinal device capable of sensing and encoding optical stimuli, achieving over 96% inference accuracy in image recognition tasks^30^. However, polarization-sensitive devices capable of multi-dimensional input control, encompassing gate voltage, light polarization and intensity, to enable highly efficient, compact, and versatile neuromorphic systems, remain scarcely explored.

In this work, we manufacture an intrinsically anisotropic black arsenic-phosphorus (b-As_0.2_P_0.8_)-based device capable of extending the capabilities polarization-sensitive photoreceptors, leveraging multi-dimensional inputs, including polarization and gate voltage, in the human retina at the near-infrared communication band. Basic polarization-sensitive photoresponse behavior is probed with a laser source of 1550 nm, where a record high polarization ratio of 4.66 is observed with an incident light power density of 0.04 W mm^−2^. Such sensitive photoresponse enables the applications of the device as synapse with low energy consumption ~25 pJ per spike. Using these neuromorphic responses, we developed a hybrid optical-electronic neural network (HOENN) capable of performing classification and reconstruction tasks, achieving over 90% classification accuracy on the Fashion-MNIST dataset and 71.38% reconstruction accuracy on the Yale Face Database under linear polarization. Additionally, our phototransistor, based on programmable photosensitivity, exhibits high-resolution polarization-sensitive imaging reconstruction of light polarization and intensity information, which provides a promising platform for compact, miniaturized and multifunctional photodetectors.

## Results

### Anisotropic characterization of high-quality b-As_0.2_P_0.8_ crystals

Figure 1a illustrates the biological foundation of neuromorphic vision sensors, where presynaptic neurons release neurotransmitters in response to external stimuli, triggering excitatory or inhibitory postsynaptic currents (PSCs) in postsynaptic neurons through mechanisms regulated by synaptic plasticity, which enables adaptive learning, dynamic signal modulation, and long-term memory formation for complex visual processing. However, the human visual system, despite its remarkable capabilities, remains largely insensitive to polarization—a critical optical parameter that encodes material anisotropy, surface orientations, and scattering properties—thereby limiting its ability to fully extract and utilize polarization-specific information inherent in natural and artificial environments. To address the challenge, the biologically inspired neuromorphic framework emphasizes the need for advanced materials capable of mimicking synaptic behaviors, positioning anisotropic materials b-As_0.2_P_0.8_ as an ideal candidate for realizing polarization-sensitive functionalities. As shown in Note S1 and Fig. S1, the alloy films obtained by our modified growth method exhibit a flat surface with a low roughness of 0.6 nm, single-crystal characteristics, and homogeneous alloy composition. The films were able to form centimeter-scale, highly oriented b-As_x_P_1-x_ flakes with well-defined shapes and smooth surfaces, indicating reduced defect density. Figure S2a presents the b-As_0.2_P_0.8_ crystal structure from front, side, and top views, revealing an orthorhombic lattice with a puckered honeycomb configuration formed by covalently bonded arsenic and phosphorus atoms within each atomic layer, where pronounced in-plane anisotropy manifests as distinct armchair (AC) and zigzag (ZZ) directions. The calculated band structure along the X-S-Y-Γ-X path of the Brillouin zone, shown in Fig. 1b, reveals that the Γ-X and Γ-Y directions correspond to the AC and ZZ crystallographic axes in real space, aligning with the material’s anisotropic nature. The results confirm that b-As_0.2_P_0.8_ is a direct bandgap semiconductor with both the conduction band minimum (CBM) and valence band maximum (VBM) located at the Γ point, yielding a bandgap of approximately 0.28 eV. The partial density of states analysis, as illustrated in Fig. S2b, demonstrates that the 3p orbital electrons in the black phosphorus structure exhibit strong contributions near the Fermi level, while the influence of 3 s orbital electrons is relatively weak. However, a notable shift is observed as the arsenic content increases, wherein the P-3p orbitals contribute progressively less near the Fermi level, while the As-4p orbitals become increasingly dominant, thereby revealing a composition-dependent electronic reconfiguration that underpins the tunability of the material’s electronic properties for polarization-sensitive optoelectronic applications. To further elucidate the anisotropic electronic properties of the b-As_0.2_P_0.8_ crystal, the partial charge density distributions corresponding to the CBM and VBM were calculated, as shown in Figure S2c, wherein the spatial wavefunction distributions exhibit pronounced directional asymmetry along the crystallographic a-axis and b-axis, a feature that is intrinsically linked to the puckered honeycomb lattice structure and serves as the primary origin of the b-As_0.2_P_0.8_ in-plane optical anisotropy. Polarization-resolved absorption spectra of b-As_0.2_P_0.8_, acquired via Fourier-transform infrared (FTIR) microscopy in Fig. 1c, performed on crystals transferred onto CaF_2_ substrates, reveal pronounced polarization-dependent absorption along the parallel crystal axis, confirming the robust anisotropic infrared optical absorption. The anisotropic vibrational properties of b-As_0.2_P_0.8_ were investigated using angle-resolved polarized Raman spectroscopy with a linearly polarized incident laser, as shown in Fig. 1d, e. At any polarization angle, the Raman spectrum of b-As_0.2_P_0.8_ (Fig. 1d) exhibits three prominent peaks at 361, 435, and 460 cm^−1^, which are quantitatively consistent with values reported in the literature^31^, confirming the reliability of the observed vibrational modes and their correlation with the crystal’s anisotropic lattice structure. As shown in Fig. 1f (i-iii), the intensities of the Raman peaks exhibit distinct variations with the rotation of the laser polarization direction, revealing a twofold symmetry for the *A*_g_^1^mode and a fourfold symmetry for the *A*_g_^2^ and *B*_2g_ modes, which underscores the intrinsic anisotropic vibrational properties.

### Phototransistor configuration and polarization-dependent performance

**Fig. 2:**
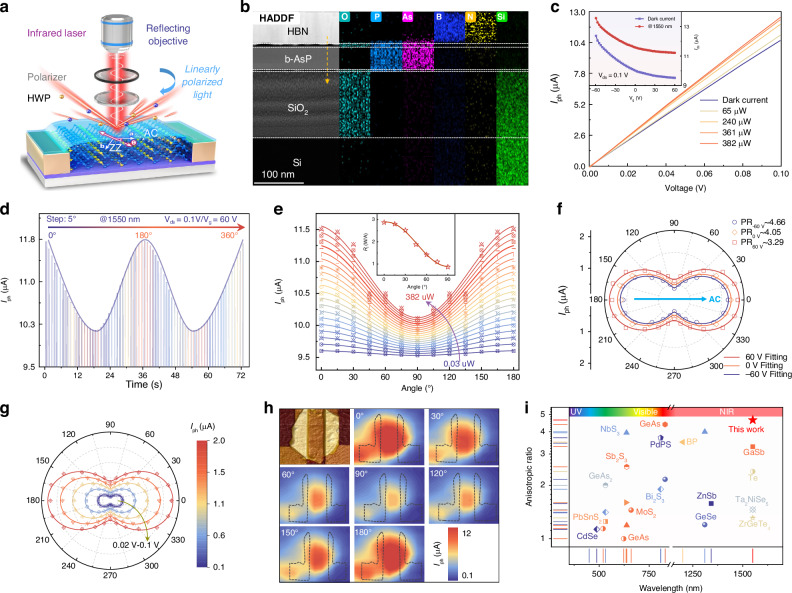
Polarization photoelectric performance of b-As_0.2_P_0.8_ phototransistor. **a** Schematic structure of the b-As_0.2_P_0.8_ polarization phototransistor. **b** Cross-sectional TEM image and the high-angle annular dark field (HAADF) of the device fabricated on a SiO_2_/Si substrate, and the element distributions. **c** Output characteristic *I*_ph_-*V*_ds_ curves under 1550 nm laser irradiation at different powers, with the inset depicting the transfer curves under dark and illumination at fixed *V*_ds_ = 0.1 V. **d** Anisotropic total current of the device under linearly polarized 1550 nm laser illumination. **e** The anisotropic response of the net photocurrent as incident power (*P*_in_ from 0.03 to 382 μW) demonstrates a stable anisotropic behavior. **f** The polar plot showing angle-resolved photocurrent for the *V*_g_ of −60, 0, and 60 V, revealing periodic variations consistent with the inherent anisotropy. **g** The polar plot shows the angle-resolved photocurrent for *V*_ds_ ranging from 0.02 V to 0.1 V. **h** Spatial mapping of the photocurrent across the b-As_0.2_P_0.8_ phototransistor under different polarization orientations of the incident laser, recorded at *V*_ds_ = 0.1 V and *V*_g_ = 60 V. The photocurrent reaches a maximum when the light polarization is aligned at 0° and a minimum at 90°, emphasizing the polarization sensitivity of the device. **i** Comparison of the photocurrent anisotropic ratio and photoresponse range of the b-As_0.2_P_0.8_ phototransistor against those of other widely used 2D and 1D materials^14,46–48^

A typical phototransistor was fabricated using a back-gated field-effect architecture with mechanically exfoliated b-As_0.2_P_0.8_ nanosheets oriented along the AC direction, as illustrated in Fig. 2a. The thickness of the b-As_0.2_P_0.8_ nanosheets was determined to be ~40 nm (Fig. S4), which was selected as the optimal thickness to balance photoresponse and dark current. The atomic-scale single-crystallinity of the phototransistor was rigorously validated through scanning transmission electron microscopy (STEM), where aberration-corrected imaging (Fig. 2b) provided a pristine cross-sectional view of the device, confirming the absence of external contamination and oxide layers that could compromise device performance. High-resolution transmission electron microscopy (HRTEM) (Fig. S5a–c) revealed a defect-free lattice arrangement of As and P atoms with precise interplanar spacings of 3.4 Å and 4.7 Å along the (101) and (010) planes, respectively. The spacing observed along the AC direction compared to the ZZ direction aligns with the greater atomic radius of As relative to P, as corroborated by XRD results (Fig. S3c). In addition, energy-dispersive X-ray Spectroscopy (EDS) mapping (Right panel of Fig. 2b) confirmed the uniform distribution of As, P, Si, O, B, and N throughout the cross-section, establishing the compositional homogeneity and structural integrity of the synthesized b-As_0.2_P_0.8_ crystal. The results show that the exfoliation of b-As_0.2_P_0.8_ and subsequent device fabrication, performed in an ambient environment, leads to the formation of an amorphous native phosphorus oxide layer (2–4 nm thick) on both the top and bottom surfaces of the flakes. The presence of these phosphorus oxide layers on both (top and bottom) surfaces of the b-As_0.2_P_0.8_ flake is expected to induce defects. The h-BN encapsulation primarily functions to prevent further environmental oxidation. This intrinsic oxide layer, in turn, creates carrier-trapping states that prolong charge carrier lifetime, thereby facilitating synaptic behaviors. Figure 2c presents the typical photocurrent-bias voltage (*I*_ph_-*V*_ds_) characteristics of the b-As_0.2_P_0.8_ phototransistor under 1550 nm laser illumination, with incident light intensities varying from 0 to 382 μW, and the polarization angle aligned along the AC direction. The contact barrier height of the b-As_0.2_P_0.8_/Au interface was previously determined to be 35 meV, suggesting that the contribution of the Schottky barrier to the photovoltaic effect is negligible^32^. The inset of Fig. 2c shows the transfer characteristics of the device, demonstrating a clear p-type field-effect behavior, further confirming the effective modulation of the channel current by the gate voltage (*V*_g_) under both dark and illuminated conditions, where drain current decreases with increasing gate voltage. By dynamically modulating the laser on-off state and systematically adjusting the polarization angle using a half-wave plate, the photocurrent of device was recorded, as shown in Fig. 2d, where the photocurrent exhibits distinct periodic variations with a maximum along the AC-axis and a minimum along the ZZ-axis of the b-As_0.2_P_0.8_ nanosheets, and the polarization ratio (PR = *I*_ph,max_/*I*_ph,min_) is 3.29.

The photocurrent signals are consistent with the inference in Fig. 2c and support the mechanism that the photoconductive effect dominates at bias mode, eliminating the influence of electrode-induced Schottky barrier effects. The spatial mapping of photocurrent across the channel, shown in Fig. 2h, reveals a clear polarization-sensitive response, where the photocurrent varies periodically with the incident light polarization angle^33^. Note S4 and Fig. S6 provide more detailed discussion of the working mechanism and energy band diagrams under different conditions. Figure 2e–g illustrate the polarization-dependent photocurrent characteristics of the b-As_0.2_P_0.8_ phototransistor under varying *P*_in_, *V*_ds_, and *V*_g_. The b-As_0.2_P_0.8_ phototransistor further demonstrates its excellent broadband response capability across the 0.52–4 μm range in Fig. S7a, b. Figure 2e shows the photocurrents as a function of polarization angles under various incident intensities from 0.03 to 382 μW. The photocurrent exhibits a clear periodic modulation, with maximum values along the AC-axis (0°) and minimum values along the ZZ-axis (90°), reflecting the intrinsic in-plane optical anisotropy of the material. The inset of Fig. 2e further quantifies the device’s responsivity (*R*_I_) as a function of the polarization angle, showing a similar periodicity that aligns with the photocurrent variations with a peak responsivity of 2.88 A W^−1^, which is superior to that of both the latest and typical 2D material infrared detectors (Fig. S7c and Table S1). The polarization-resolved photocurrent measurements under the different *V*_g_ (−60, 0, and 60 V) are plotted in Figs. 2f and S7d–f, which fits the function equation^34^:

1 $${I}_{{ph}}={I}_{{ph},\mathrm{AC}}{\cos }^{2}(\varphi )+{I}_{{ph},\mathrm{ZZ}}{\sin }^{2}(\varphi )$$

with *I*_ph,AC_ and *I*_ph,ZZ_ representing the photocurrent components along the AC and ZZ directions, respectively. The results achieved a large PR of about 4.66, underscoring the pronounced optical anisotropy inherent to the b-As_0.2_P_0.8_ crystal. Figure 2g presents the polar plot of the photocurrent as a function of the polarization angle under varying bias (−0.1, 0, and 0.1 V), forming distinct two-lobed patterns that reflect the strong in-plane optical anisotropy of the device. These results confirm the tunability of polarization sensitivity in the b-As_0.2_P_0.8_ phototransistor through applied *P*_in_, *V*_ds_, and *V*_g_, enabling dynamic control of anisotropic photoresponse, which is essential for advanced polarization-sensitive infrared imaging and optoelectronic applications requiring adaptive polarization discrimination. Compared to carbon nanotube^35^ and CdSe^36^ nanowire photodetectors, which necessitate complex nanofabrication, the b-As_0.2_P_0.8_ phototransistor leverages its intrinsic crystal anisotropy for robust linear dichroism detection. Figure 2i summarizes the photoresponse range and anisotropic ratios of prominent 2D and 1D materials, highlighting the optimal polarization-sensitive performance of the b-As_0.2_P_0.8_ phototransistor in the IR regime.

### Emulation of polarization-sensitive b-As_0.2_P_0.8_ synaptic phototransistor

**Fig. 3:**
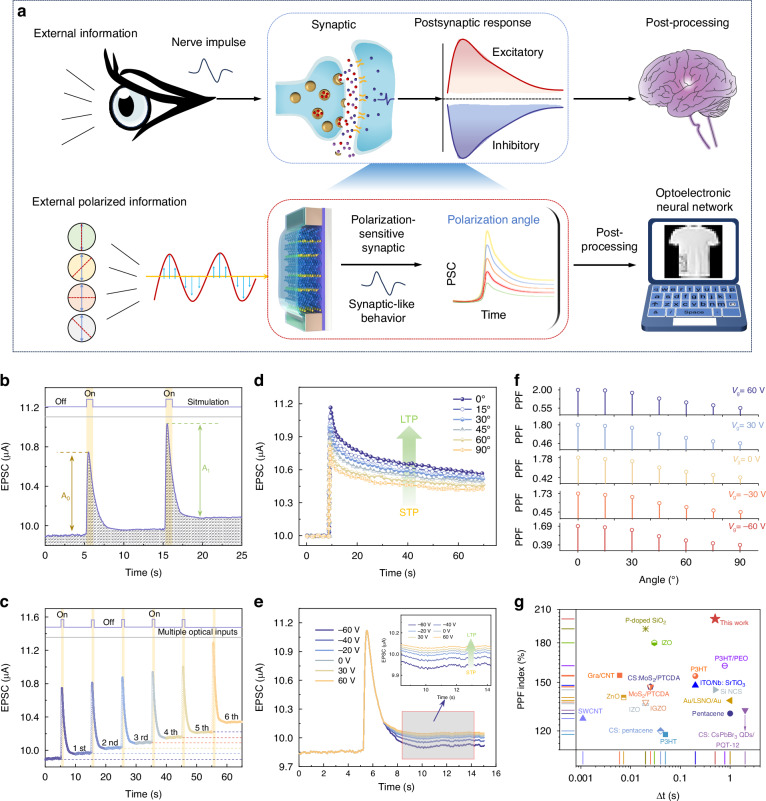
Electrical and optical stimulation of synaptic functions of b-As_0.2_P_0.8_ phototransistor. **a** Schematic diagram comparing biological synapses and designed artificial polarization synaptic devices. **b** Changes in EPSC induced by paired presynaptic optical pulses with a 1 s pulse width. *A*_0_ and *A*_1_ denote the changes in EPSC observed after the first and second pulses, respectively. **c** Transition of EPSC from ST to LTP through six repeated stimulations under 1550 nm illumination. **d**, **e** EPSC response as a function of varying polarization angles and *V*_g_, respectively. **f** PPF index of b-As_0.2_P_0.8_ device under different polarizations with *V*_g_ from −60 to 60 V under 1550 nm laser. **g** Statistical comparison of PPF ratios in the b-As_0.2_P_0.8_ device relative to similar previously reported devices^49,50^

Figure 3a illustrates the conceptual framework of polarization-sensitive neuromorphic vision sensing, drawing inspiration from biological neural systems. In the upper panel, the biological process begins with external visual stimuli generating nerve impulses, which are transmitted to synapses where neurotransmitters mediate the postsynaptic response. The lower panel of Fig. 3a extends this bioinspired paradigm to the optoelectronic domain, where external polarized light information is detected and processed by the polarization-sensitive b-As_0.2_P_0.8_ synaptic phototransistor. Mimicking the postsynaptic currents (PSC) dynamics observed in biological synapses, the device utilizes the polarization angle of incident light to modulate the photocurrent magnitude, enabling real-time, polarization-resolved signal processing that cannot be achieved by natural visual systems. The bioinspired yet fundamentally enhanced approach bridges the gap between biological and artificial systems, offering a scalable platform for advanced neuromorphic vision and adaptive polarization-sensitive applications. The channel conductivity (synaptic weight) of phototransistor is critical for information processing in these networks. To emulate the synaptic connection, we define the device’s drain and source terminals as presynapse and postsynapse, respectively, with the gate terminal and polarization state functioning as the modulatory inputs. The phenomenon of paired-pulse facilitation (PPF)^37^, a temporary information encoding mechanism in biological synapses, can be observed in the b-As_0.2_P_0.8_ device when consecutive excitatory postsynaptic currents (EPSC) are generated by two closely spaced optical spikes. Figure 3b illustrates the typical EPSC of the polarization-sensitive b-As_0.2_P_0.8_ synaptic phototransistor under 1550 nm illumination (400 μW, 1 s) with a *V*_ds_ of −0.1 V. The initial spike (*A*_0_) reflects the optical response induced by the first light pulse, while the second spike (*A*_1_) exhibits a significantly higher EPSC magnitude due to the residual photogenerated carriers from the first stimulation. These carriers, which do not fully recombine within 1 s, accumulate and contribute to the increased photocurrent, effectively strengthening the synaptic weight. This slow recovery behaviour is attributed to recombination processes mediated by localized trap states introduced by surface oxidation. Specifically, this oxidation creates electron trap states at the semiconductor-dielectric interface that acts as long-lived charge traps, prolonging the decay of photogenerated carriers^38,39^. Under 1550 nm illumination, photogenerated electron-hole pairs form, with holes enhancing channel conduction while electrons can be captured by oxide-related traps. Figure 3c extends this behavior to multiple optical stimulations at 1550 nm, illustrating a progressive enhancement in EPSC amplitudes with successive pulses as photogenerated carriers accumulate due to incomplete recombination.

In biological systems, synaptic plasticity is the basis of memory and learning processes categorized into short-term plasticity (STP) and long-term plasticity (LTP), where STP maintains transient information, and repeated reinforcement transfers selected information into LTP to facilitate learning and decision-making processes. In Fig. 3c, 1550 nm optical spikes with a frequency of 0.1 Hz generate cumulative EPSC due to the accumulation of the residual photogenerated carriers in response to each stimulus, illustrating STP characteristics. Figure 3d demonstrates the influence of polarization angle on EPSC retention. The b-As_0.2_P_0.8_ synaptic phototransistor emulates this neuroplasticity, enabling a transition from STP to LTP under controlled optical and electrical modulations in Fig. 3d. As the polarization angle transitions from 0° to 90°, the EPSC gradually decreases, indicating a polarization-sensitive synaptic response, effectively mimicking biological LTP behavior and allowing precise control of synaptic weights via polarization angle. Figure 3e explores the effect of *V*_g_ on synaptic behavior, where increasing *V*_g_ from −60 to 60 V results in a progressive enhancement of EPSC retention and facilitates the STP-to-LTP transition. The inset reveals that higher gate voltages prolong retention time by modulating carrier recombination dynamics, further reinforcing the synaptic weight. Specifically, negative gate voltages (−60 V to −20 V) produce pronounced signal decay after stimulation (STP behavior), while positive gate voltages (especially +30 V, +60 V) show diminished decay, transitioning to LTP-like behavior with minimal signal loss. At negative gate voltages, upward band bending accumulates holes in the channel and simultaneously reduces the energy barrier for photogenerated electrons to become trapped at surface defects. These trapped electrons screen hole carriers, causing the observed current decay. At positive gate voltages, downward band bending depletes holes from the channel but also raises the energy level of trap states relative to the conduction band, reducing the barrier for electron release. This field-assisted de-trapping mechanism explains the diminished signal decay observed at positive gate biases. We further extracted the PPF index under different *V*_g_ (−60 to 60 V) and polarization angles (0° to 90°) in Fig. 3f to support this model, showing stronger facilitation at positive gate voltages across all polarization angles, indicating enhanced de-trapping under positive bias conditions. These results demonstrate that both polarization angle and gate voltage can serve as effective external control parameters to emulate adaptive learning behaviors, enabling the b-As_0.2_P_0.8_ synaptic device to achieve tunable neural plasticity for neuromorphic computing applications. The power consumption is a critical parameter in evaluating synaptic device performance. It can be calculated using the following formula^40^:

2 $${\int }_{t0}^{t1}{V}_{{ds}}\cdot {I}_{{ph}}(X){dx}$$

where t_0_ and t_1_ represent the time of light on and light off, respectively. By utilizing a pulse width of 0.5 s and applying a *V*_ds_ of 0.01 V under 1550 nm illumination, the *I*_ph_ can be reduced to approximately 5 nA, and the energy consumption per spike can be as low as 25 pJ, which is lower than typical electric memristor (~1 nJ spike^−1^)^41^. The PPF index illustrates the capability of the polarization-sensitive b-As_0.2_P_0.8_ synaptic phototransistor to emulate short-term synaptic plasticity observed in neuroscience, calculated as the following equation:

3 $${PPF}=\frac{{A}_{1}}{{A}_{2}}\times 100 \%$$

The maximum PPF index reached 201%, highlighting the robust synaptic plasticity of the device. Figure 3g compares the PPF index and time interval (Δt) of the b-As_0.2_P_0.8_ synaptic phototransistor with a range of previously reported materials and devices, including 2D materials (e.g., MoS_2_, ZnO), organic semiconductors (e.g., P3HT, pentacene), and perovskite-based devices, where the b-As_0.2_P_0.8_ device demonstrates a superior PPF index of over 200% at intermediate retention times, outperforming most traditional materials in its ability to sustain synaptic plasticity with enhanced temporal dynamics. Table S2 summarizes the performance comparison with state-of-the-art polarization-sensitive neuromorphic devices.

### Neuromorphic computing simulation and polarization imaging application

**Fig. 4:**
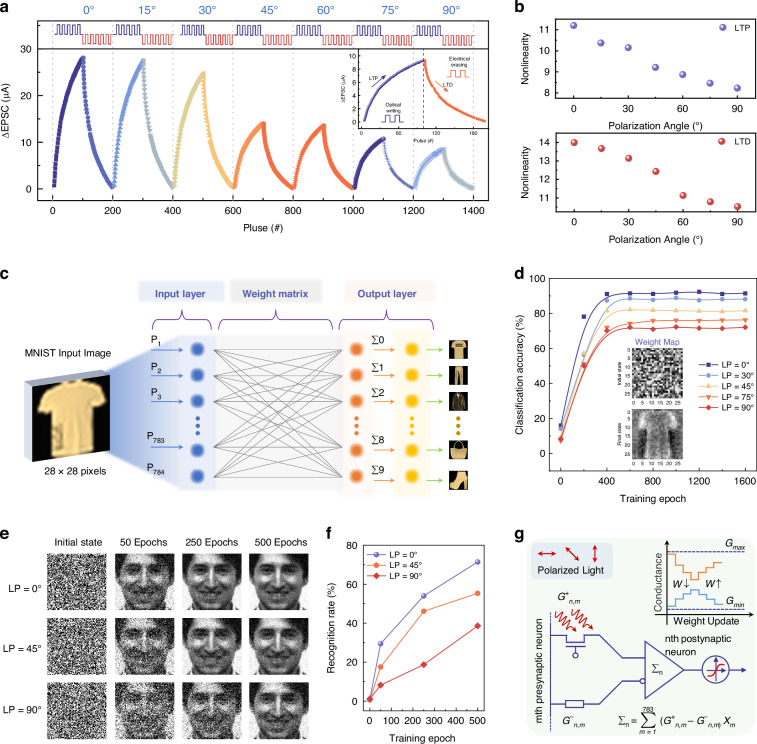
Neuromorphic computing simulation for classification and reconstruction using HOENN. **a** LTP/LTD curves under varying linear polarization states (LP = 0°–90°). **b** Calculated nonlinearity of LTP (upper panel) and LTD (lower panel) as a function of polarization angle. **c** Schematic of the simulated optoelectronic network with 28 × 28 pixels input image from the Fashion-MNIST dataset, where the “T-shirt” category is utilized to train and test network performance. **d** Comparison of classification accuracy rates across multiple polarization angles (0°, 30°, 45°, 75°, and 90°) of the LTP/LTD pulses. The inset shows a representative input category mapping of the 784 synaptic weights to the output ‘T-shirts’ shown at the initial and final states of training. **e** The reconstruction results of the input image across training cycles (initial, 50, 250, and 500 epochs), with each cycle’s LTP/LTD pulses having different polarization angles (0°, 45°, and 90°). **f** Reconstruction accuracy as a function of training epochs for LP = 0°, 45°, and 90°. **g** Synaptic weight calculations, represented as the difference between device conductance (*G*^+^_n,m_) and conductance normalizing factor (*G*^-^_n,m_), to illustrate the underlying learning dynamics, the inset shows the bidirectional weight update method

Polarization-sensitive neuromorphic synapses replicate the plasticity of biological synapses, enabling the perception, weighting, and memory of light polarization signals as the fundamental functional units of polarization neural networks. The resulting neural networks, formed by the collaborative operation of multiple synapses, integrate outputs from multiple synapses to perform intelligent information processing tasks including image classification and reconstruction. Polarization imaging directly utilizes the polarization-sensitive properties of these synapses to extract target polarization features through Stokes parameter reconstruction. Together, these three components establish a complete functional chain for polarization vision, progressing systematically from single-synapse perception, through network-based intelligent processing, to imaging applications. To investigate the long-term plasticity of our b-As_0.2_P_0.8_ device for pattern reconstruction potential, we first analyzed its weight update dynamics. Long-term plasticity includes both long-term potentiation (LTP) and long-term depression (LTD), with LTD likely reversing the effects of prior LTP. Consequently, the balance between LTP and LTD maintains synaptic weights within a linear range, allowing for the agile processing of frequency-based signals. As shown in Fig. 4a we applied a sequence of 100 continuous light pulses (1550 nm, width = 1 s, interval = 1 s) at various linear polarization angles (LP = 0°–90°), followed by 100 continuous electrical pulses (*V*_g_ = −60 V, width = 1 s, interval = 1 s) to obtain the conductivity (weight) update trajectory. Under light pulses, the device’s conductance increased, demonstrating LTP, while electrical pulses induced LTD. These optoelectronic pulse interactions mimic the synaptic weight updates in biological neural networks. Unlike memristors that rely solely on electric pulses, our b-As_0.2_P_0.8_ devices natively encode two-dimensional information using polarized light inputs. The polarization angle modulates synaptic weight via photoconductance, light intensity controls input signal magnitude, and gate voltage governs electrical erasing. This direct polarization-to-weight mapping eliminates the need for external polarizers and digital pre-processing, simplifying the system compared to traditional electric-based approaches. Utilizing this property, we mapped device conductance to simulate neural network weights, enabling us to construct a hybrid optoelectronic neural network (HOENN), with detailed principles provided in the “Methods” and Fig. S8. For optimal network performance and training speed, two parameters are crucial: the weight variability range, expressed as *G*_max_/*G*_min_, and the weight update speed, denoted by the nonlinearity factor (NL) of the LTP/LTD curves (specifically *β*_p_ and *β*_d_). Drawing from previous studies^42^, we derived expressions for LTP and LTD weight updates:

4 $$G(t)={G}_{{\min }}+\frac{{G}_{{\max }}-{G}_{{\min }}}{{\beta }_{p}}\,\mathrm{ln}\left[\frac{{\beta }_{p}({\alpha }_{p}t+{C}_{p})}{{G}_{max}-{G}_{min}}\right]$$

5 $$G(t)={G}_{{\min }}+\frac{{G}_{{\max }}-{G}_{{\min }}}{{\beta }_{d}}\,\mathrm{ln}\left[\frac{{\beta }_{d}({\alpha }_{d}t+{C}_{d})}{{G}_{max}-{G}_{min}}\right]$$

In which the undetermined coefficients are *α*_p_ and *α*_d_. While *C*_d_ is a constant related to the starting point of timing, and does not affect the trend of the curve. In Fig. 4b, we present fitted NL values across polarization states using experimental data and training flowchart (see Note S5, Figs. S9 and S10 for details). The results demonstrate that linear polarization at 0° (LP = 0°) yields the highest NL value, attributed to the widest conductance variation range and the fastest rate of change observed under LP = 0° conditions. Table S3 summarizes the extracted LTP/LTD parameters under different polarization angles from experimental data.

For our supervised learning task, illustrated in Fig. 4c, we constructed a HOENN to classify data from the Fashion-MNIST dataset, a more challenging benchmark than MNIST, used in previous LTP/LTD neural network studies^43^. Each Fashion-MNIST image (28×28 pixels) was flattened into a 784-dimensional vector, each representing an input layer node. The output layer contained 10 nodes, corresponding to categories like ‘T-shirt’, ‘Dress’, ‘Bag’, and others. The network’s output is defined as the following equation:

6 $$y=\sigma ({Wx}+b)$$

where σ is the sigmoid function, W is the 784 × 10 weight matrix, x is the 784-dimensional input vector, b is the bias term, and y is the 10-dimensional output vector. Cross-entropy loss and backpropagation were used for weight updates. In Fig. 4d, we analyze HOENN’s classification accuracy across polarization states, where training for approximately 400 epochs yielded an accuracy exceeding 90% under LP = 0°. Notably, this accuracy is achieved on a more complex dataset compared to previous studies with similar topologies^44,45^. The inset in Fig. 4d illustrates weight visualizations for the “T-shirt” category node before and after 800 training iterations, revealing alignment with the “T-shirt” pattern, which enables larger output values and correct classification. These visualized weights, representing a generalized feature map for the image class rather than an individual image, underscore the effectiveness of the training (Fig. S11 and Note S6). In an unsupervised learning experiment, we tested HOENN in Encoder-Decoder architecture (dimensions 10000–2500–10000, Fig. S12) to reconstruct images from the Yale Face Database, using each input image as its target. Training minimizes mean squared error between outputs and original pixel values. Figure 4e shows HOENN’s reconstruction performance across polarization states (LP = 0°, 45°, and 90°) for the same image after various training epochs. To objectively assess the fidelity of the reconstructed images, we used the Structural Similarity Index Metric (SSIM). As shown in Fig. 4f, after 500 epochs, HOENN under LP = 0° achieved a reconstruction accuracy of 71.38%, outperforming other polarization states. This demonstrates that polarization-state encoding strategies can fully exploit the dynamic response range of devices, thereby expanding the regulatory dimensions of weight parameters. Figure 4g demonstrates our approach to synaptic weight calculation by using the differential conductance (*G*^+^_n,m_, *G*^−^_n,m_), as well as a schematic diagram of the bidirectional weight update method, offering key insights into HOENN’s operation. Detailed weight update algorithms and SSIM definitions are available in Note S7. This dual-device topology enables precise differential weight control, surpassing the linearity limitations of single-memristor systems.

**Fig. 5:**
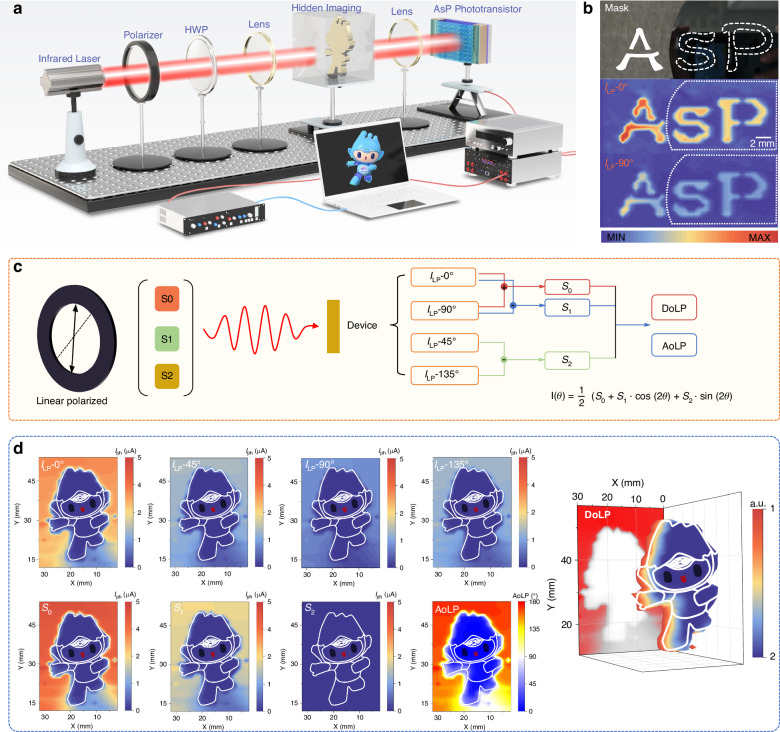
Multifunctional polarization imaging of the b-As_0.2_P_0.8_ phototransistor. **a** Schematic diagram of the 2D scanning imaging system. **b** Semi-concealed imaging of the letters ‘AsP’ under parallel and perpendicular polarizations at 1550 nm illumination, where the high-resistance silicon wafer obscures “sP”. **c** The Stokes parameter calculation based on the b-As_0.2_P_0.8_ device. **d** Linear polarization imaging with four different polarization angles 0°, 45°, 90°, and 135° and the DoLP imaging results of the device. All demonstrations were performed at the 1550 nm laser

To demonstrate the polarization imaging capabilities of the b-As_0.2_P_0.8_ phototransistor, a polarization-resolved infrared imaging system was constructed, as shown in Fig. 5a, incorporating a 1550 nm infrared laser source, a polarizer, a half-wave plate (HWP), focusing lens, and the b-As_0.2_P_0.8_ phototransistor as the detection element with the target object positioned on a 2D translation stage. The phototransistor converts optical signals into electrical signals, which are then displayed as grayscale values on the screen. Specifically, imaging was achieved by raster-scanning a focused 1550 nm laser spot across a stationary mask. Stokes parameters were computed at each scan position using the photocurrents from b-As_0.2_P_0.8_ phototransistor (see Note S8 for further details). The interaction of the polarized light with the HWP in the system can be described using the Jones matrix formalism:

7 $${E}_{{out}}={M}_{{HWP}}\left(\theta \right)\cdot {E}_{{in}}$$

where *E*_in_ and *E*_out_ are the Jones vector of the incident and outgoing light, and *M*_HWP_(*θ*) is the Jones matrix of the half-wave plate. The system relies on the polarization sensitivity of the device to capture spatially resolved photocurrent responses under varying linear polarization angles (*I*_LP-θ_) and reconstruct detailed polarization information of the hidden imaging target. High-resistance silicon wafers exhibit over 80% transmittance (Fig. S13), underscoring the potential for hidden imaging applications. As depicted in Fig. 5b, under 1550 nm illumination, the high-resistance silicon wafer partially semi-hides the letter “sP” while leaving the letter “A” visible, producing distinct brightness contrasts and clear polarization-dependence in the parallel and perpendicular directions. The polarization-resolved intensity maps (*I*_LP-0°_ and *I*_LP-*9*0°_) of hidden objects through silicon wafer reveal distinct features of the target that are invisible under unpolarized illumination.

The characterization of polarization states is grounded in the Stokes formalism, as illustrated in Fig. 5c, where Stokes parameters (*S*_0_, *S*_1_, *S*_2_) are extracted from photocurrent measurements. The general form of Stokes vector ***S*** is given by:

8 $${\rm{S}}=\left[\begin{array}{c}{S}_{0},\, {S}_{1},\, {S}_{2},\, {S}_{3}\end{array}\right]^T$$

where *S*_3_ describes the circular polarization component, which is negligible for linearly polarized light in this study. The photocurrent at a given polarization angle θ can be expressed as:

9 $$I(\theta )=\frac{1}{2}({S}_{0}+{S}_{1}\cdot \cos \left(2\theta \right)+{S}_{2}\cdot \sin \left(2\theta \right))$$

representing the decomposition of light into its horizontal (*S*_1_) and diagonal (*S*_2_) polarization components (see Note S8 for more calculation process).

Figure 5d demonstrates the polarization-resolved imaging capabilities of the b-As_0.2_P_0.8_ phototransistor through spatially reconstructed intensity maps and Stokes parameter analysis. The four subplots in the top row show the polarization-resolved imaging distributions of the Hangzhou Asian Games mascot hidden in a high-resistivity silicon box (*I*_LP-0°_, *I*_LP-45°_, *I*_LP-90°_, and *I*_LP-135°_) measured under linear polarized light at respective angles. The second row presents the derived Stokes parameters (*S*_0_, *S*_1_, and *S*_2_), which collectively provide a comprehensive mathematical representation of the target’s polarization state, where *S*_0_ represents the total intensity of polarized light across all angles, effectively delineating the overall shape and structural features of the mascot, while *S*_1_ quantifies the relative contributions of horizontal (0°) and vertical (90°) polarization components to highlight dominant polarization directions in specific regions, and *S*_2_ captures the diagonal (45°and 135°) polarization components, thereby enriching the depth and detail of the polarization analysis and enabling precise spatial characterization. The final two panels illustrate the derived polarization metrics, including the degree of linear polarization (DoLP) and the angle of linear polarization (AoLP), where the DoLP map, generated under a 1550 nm imaging wavelength, reveals regions of strong polarization contrast with higher DoLP values highlighting areas exhibiting well-defined polarization states, effectively uncovering intricate structural details of the Asian Games mascot that are invisible in conventional intensity-based imaging. The AoLP map encodes the angular orientation of the polarized light at each spatial location, providing critical directional information for characterizing the complex optical properties of the mascot. In addition, Figs. S14 and S15 show the linear polarization imaging of “hang” and “AsP” with four different polarization angles 0°, 45°, 90°, and 135° and the DoLP imaging of the device. The three-dimensional representation of the DoLP further accentuates the spatial distribution of polarization sensitivity, underscoring the capability of the b-As_0.2_P_0.8_ phototransistor to capture and reconstruct polarization-resolved information with high fidelity and spatial resolution, enabling the precise detection of subtle features or hidden patterns in optically challenging environments. The current device is optimized for linear polarization imaging (*S*_0_, *S*_1_, and *S*_2_). Circular polarization (*S*_3_) detection could be enabled by integrating quarter-wave plates in future designs, expanding the system’s utility for full-Stokes polarimetry (see Note S9 for further details).

## Discussion

In summary, we have demonstrated a high-performance polarization-sensitive neuromorphic vision system based on a two-dimensional b-As_0.2_P_0.8_ phototransistor, which leverages its intrinsic anisotropy and multidimensional input controls to achieve significant advancements in polarization-sensitive optoelectronics and neuromorphic computing. By coupling the strong polarization selectivity (polarization ratio of 4.7) with exceptional optoelectronic properties in the near-infrared communication band, the device mimics synaptic behaviors analogous to biological neural systems, achieving paired-pulse facilitation (PPF) values of up to 201%. These properties enable the development of a HOENN, which demonstrates superior classification accuracy (>90% on the Fashion-MNIST dataset) and remarkable reconstruction capabilities. Furthermore, we introduced a polarization-resolved imaging framework based on Stokes parameter reconstruction and DoLP mapping, capturing and visualizing hidden polarization-specific features with high spatial fidelity. This work establishes a scalable foundation for next-generation neuromorphic optoelectronic technologies, where the seamless integration of polarization sensitivity and brain-inspired computing addresses critical challenges in high-speed, low-power, and multifunctional systems. This work provides a valuable reference for developing highly integrated, low-power intelligent optoelectronic devices. By demonstrating the synergistic operation of polarization sensing, neuromorphic computing, and imaging functions at the single-device level, the study lays an experimental foundation for subsequent large-scale device integration (such as polarization-sensitive sensor arrays) and system-on-chip development. The findings not only reveal potential pathways for functional integration based on intrinsic material properties but also experimentally validate the feasibility of array-level coordinated operation through the training of hybrid optoelectronic neural networks and imaging applications. Collectively, these discoveries offer critical insights for advancing the practical deployment of brain-inspired optoelectronic technologies in real-world scenarios.

## Materials and methods

### Fabrication of phototransistors

Cr (5 nm)/Au (50 nm) drain and source electrodes were patterned on 300 μm-thick Si substrates using standard UV lithography, followed by SiO_2_ (500 nm) deposition. Electron beam evaporation in vacuum (<5 × 10^−4^ Pa) and a lift-off process in acetone were employed to define the electrodes. B-As_0.2_P_0.8_ was encapsulated with boron nitride to preserve its intrinsic properties. Si/SiO_2_ substrates and boron nitride crystals were supplied by our collaborators Shanghai Onway Technology Co., Ltd.

### Characterization and photoelectric testing of phototransistors

Optical images were captured with an Olympus DSX1000 optical microscope, while Raman spectra were acquired using a Renishaw inVia micro-Raman system with a 532 nm laser. AFM imaging was conducted on a Cypher ES Environment AFM. High-resolution X-ray diffraction (HRXRD) patterns were recorded on a Bruker D8 diffractometer to confirm single-crystal quality. TEM imaging of the phototransistors was performed on a Titan 80–300 TEM at 100 kV. Cross-sectional characteristics were examined using focused ion beam (FIB) preparation (FEI Nova NanoLab 600) and high-resolution scanning TEM (STEM, Thermo Scientific Themis Z, 300 kV). XPS analysis and corresponding mapping (Thermo Scientific ESCALAB Xi + ) provided detailed composition and elemental distribution for the b-As_0.2_P_0.8_ nanosheets. For spectral photoresponse measurements, b-As_0.2_P_0.8_ crystal were exfoliated onto CaF_2_ substrates, with transmission and reflection spectra collected using a Bruker FTIR spectrometer (Vertex 80 V and Hyperion 3000), referencing a pure CaF_2_ substrate and a gold mirror, respectively. The polarizer was from LBTEK. The electronic and optoelectronic properties were measured using a Lake Shore probe station and a Keithley 4200A-SCS source meter. The photocurrent mapping and polarization imaging were carried out by the MStarter 200 High Precision Photocurrent Scanning Test Microscope (Nanjing Metatest Optoelectronics Co., Ltd.).

### Using PyTorch to simulate the weight update method of HOENN

In this approach, the synaptic weights are represented by the difference in conductance between two devices, that is, *W* = *k* (*G*^+^_n,m_-*G*^−^_n,m_), where *k* denotes the amplification coefficient for the current. The changes in the required weights ΔW for each iteration are calculated using the backpropagation algorithm. When Δ*W* > 0, *G*^+^_n,m_ increases and *G*^−^_n,m_ decreases, corresponding to the photocurrent pulse, *W* ultimately increases; conversely, when Δ*W* < 0, *G*^+^_n,m_, decreases and *G*^−^_n,m_ increases, corresponding to the electric current pulse, *W* ultimately decreases.

## Supplementary information

Supplementary information

## Data Availability

The data that support the findings of this study are available from the corresponding author upon reasonable request.
