# Supplementary material for: Polarization-sensitive neuromorphic vision sensing enabled by pristine black arsenic-phosphorus
Source: Light Sci Appl. 2026 Feb 2;15:100. doi: 10.1038/s41377-025-02125-0 (PMC12865036; doi:10.1038/s41377-025-02125-0)
Supplement: Supplementary file 1 — Supplementary information [file 41377_2025_2125_MOESM1_ESM.docx]

**Polarization-Sensitive Neuromorphic Vision Sensing enabled by Pristine** **Black Arsenic-Phosphorus**

Shi Zhang^1,2,3^†, Shuguang Zhu^1^†, Shijian Tian^1^†, Libo Zhang^1,2^*, Cheng Chen^3^, Kening Xiao^1,2^, Wenqi Mo^1^, Shicong Hou^1,4^, Yunduo Zhang^1^, Yuanfeng Wen^1^, Yiran Tan^1^, Kaixuan Zhang^1^, Jiayue Han^5^, Changlong Liu^1^, Jiale He^1^*, Weiwei Tang^1^, Jun Wang^5^, Lin Wang^2^*, Guanhai Li^1,3,6^*, Kai Zhang^3^, and Xiaoshuang Chen^1,2,6^*

^1^College of Physics and Optoelectronic Engineering, Hangzhou Institute for Advanced Study, University of Chinese Academy of Sciences, No. 1, Sub-Lane Xiangshan, Xihu District, Hangzhou 310024, China.

^2^State Key Laboratory of Infrared Physics, Shanghai Institute of Technical Physics, Chinese Academy of Sciences, 500 Yu-Tian Road, Shanghai 200083, China.

^3^CAS Key Laboratory of Nanophotonic Materials and Devices & Key Laboratory of Nanodevices and Applications, i-Lab, Suzhou Institute of Nano-Tech and Nano-Bionics (SINANO), Chinese Academy of Sciences, Suzhou 215123, China.

^4^Shanghai Key Lab of Modern Optical System, University of Shanghai for Science and Technology, 516 Jungong Road, Shanghai 200093, China.

^5^School of Optoelectronic Science and Engineering, University of Electronic Science and Technology of China, Chengdu 610054, China.

^6^Suzhou Laboratory, Suzhou, Jiangsu, 215000, China

S.Z., S.G.Z., and S.J.T. contributed equally to this work.

.

**Table of contents**

**Section S1. Anisotropic Characterization of High-quality b-As_0.2_P_0.8_ Crystals**

Note S1. Synthesis instructions of b-As_x_P_1-x_ crystals.........................................................................4

Fig. S1. Growth of b-As_x_P_1-x_ crystal...................................................................................................5

Fig. S2. First principles calculation....................................................................................................6

Note S2. First principles calculation method......................................................................................7

Note S3. Characterization data details of b-As_0.2_P_0.8_ crystal...............................................................8

Fig. S3. Characterization of b-As_0.2_P_0.8_ crystal nanosheets.................................................................9

**Section S2. Phototransistor Configuration and Polarization-Dependent Performance**

Fig. S4. AFM image of the device....................................................................................................10

Fig. S5. Device element analysis and morphology characterization.................................................11

Note S4. Detection mechanism description......................................................................................12

Fig. S6. The energy band diagrams under different conditions……………………………………14

Fig. S7. Photoelectric anisotropy behavior of b-As_0.2_P_0.8_ phototransistors.......................................15

Table S1. Comparison of the *R*_I_ for different 2D photodetectors.....................................................16

Table S2. Performance Comparison with State-of-the-Art Polarization-Sensitive Neuromorphic Devices………………………………………………………………..………………………17

**Section S3. Neuromorphic Computing Simulation and Polarization Imaging Application**

Fig. S8. LTP/LTD curves of the b-As_0.2_P_0.8_ device at different electrical gate voltages.....................18

Note S5. Derivation of the relationship between LTP/LTD and time under periodic pulses..............19

Fig. S9. The fitting results of the LTP/LTD process..........................................................................20

Fig. S10. The training flowchart.......................................................................................................21

Table S3. Extracting LTP/LTD parameters under different polarization attitudes from experimental data...................................................................................................................................................22

Note S6. Weight Update method.......................................................................................................23

Fig. S11. Weight map diagram..........................................................................................................24

Fig. S12. Schematic diagram of a three-layer encoder-decoder network architecture.......................25

Note S7. Reconstruction accuracy calculation method.....................................................................26

**Section S4. Multifunctional Stokes polarization imaging**

Fig. S13. Infrared transmittance of high-resistance silicon wafer.....................................................27

Note S8. Single-device imaging mechanism………………………………………………………28

Note S9. Polarization state calculation...........................................................................................29

Fig. S14. Linear polarization imaging of “hang” ..............................................................................30

Fig. S15. Linear polarization imaging of “AsP” ...............................................................................31

**Reference**.........................................................................................................................................32

**Section S1. Anisotropic Characterization of High-quality b-As_0.2_P_0.8_ Crystals**

**Note S1. Synthesis instructions of b-As_x_P_1-x_ crystals**

The bulk b-As_x_P_1-x_ crystals, with x varying from 0 to 0.5, were synthesized via chemical vapor transport (CVT) using high-purity gray arsenic (99.9999%) and red phosphorus (99.999%) as precursors, with SnI_4_ (99.999%) and Sn (99.999%) as mineralizers (see Methods). Figure. S1a details the synthesis workflow, including the preparation of starting materials, mixture configurations, and the overall synthesis sequence. In terms of light-absorbing materials, the grown b-As_x_P_1-x_ in this work possess better crystal quality to favor the performance of the design photodetectors. All chemicals were hermetically sealed within an evacuated silica glass quartz tube, measuring 100 mm in length with an internal diameter of 85 mm. The gray arsenic and red phosphorus with molar ratios of 0:1, 1:4, 3:7, 2:3, and 1:1 was marked as bP, b-As_0.2_P_0.8_, b-As_0.3_P_0.7_, b-As_0.4_P_0.6_, and b-As_0.5_P_0.5_, respectively. The thermal treatment protocol involved ramping the temperature up to 750°C over a span of 8 hours, maintaining this peak temperature for 20 hours, subsequently reducing it to 550°C within 2 hours, and then holding it for an additional hour. The cooling process to ambient temperature was naturally conducted over 5 hours. Crystallization of b-As_x_P_1-x_ occurred at the colder end of the silica glass tubes. In the context of light-absorbing materials, the b-As_x_P_1-x_ crystals produced in this study exhibit enhanced crystal quality, which is advantageous for optimizing photodetector performance. A key innovation in this work is the application of a seed-induced growth strategy coupled with precise feed control, enabling the formation of centimeter-scale, highly oriented b-As_x_P_1-x_ flakes. Unlike the irregular, clustered morphologies typically produced by conventional methods (Figs. S1b and 1c), our synthesized b-As_x_P_1-x_ crystals exhibit well-defined shapes and smooth surfaces (Figs. S1d and 1e), indicating a reduced defect density.

**
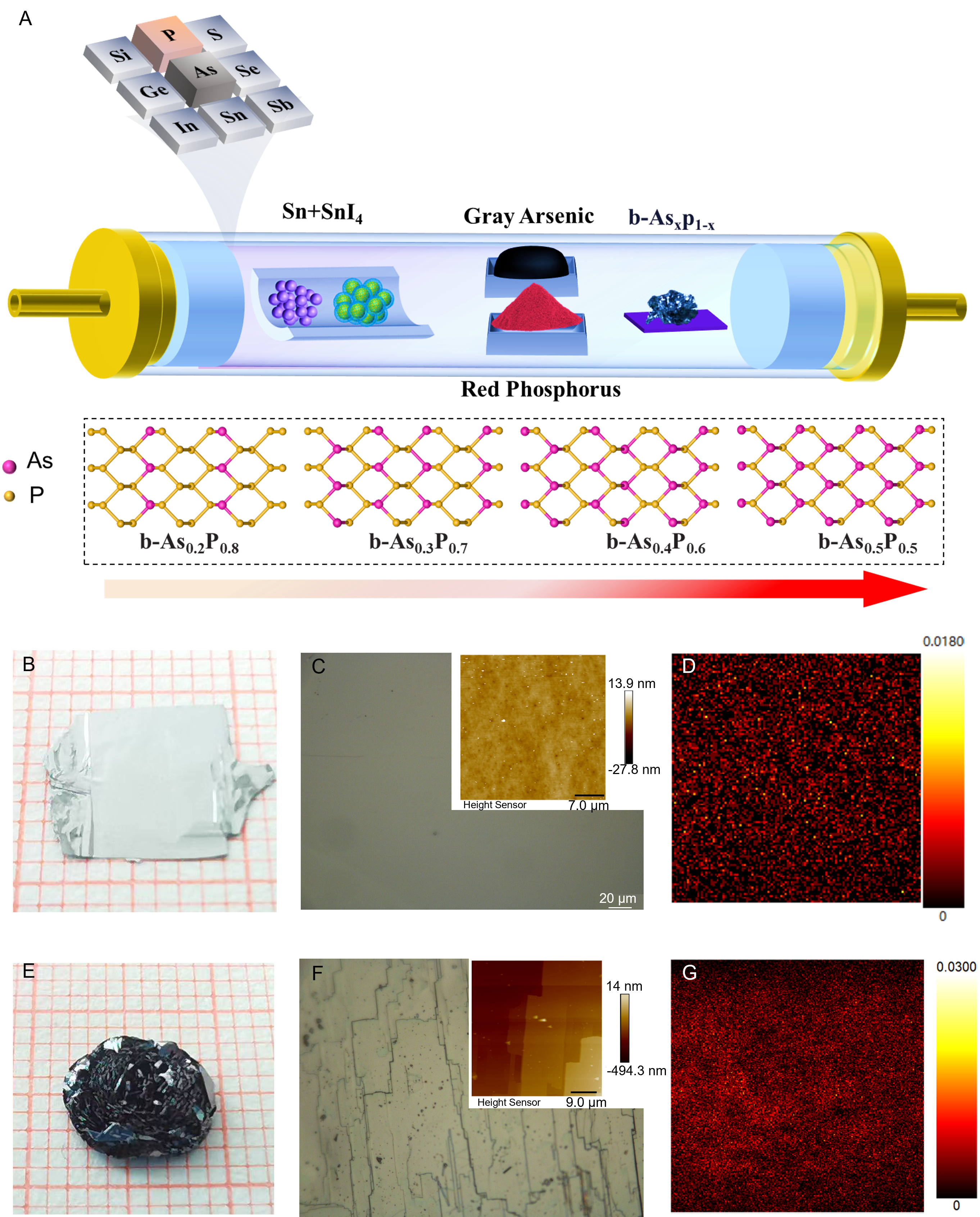
**

**Fig. S1. Growth of b-As_x_P_1-x_ crystal. a** Schematic of a typical CVD system for the synthesis of b-As_x_P_1-x_ crystal nanoplates. All chemicals were enclosed in an evacuated silica glass quartz tube (length: 100 mm; internal diameter: 85 mm). **b** A typical photograph of b-As_x_P_1-x_ crystal grown by our modified growth method. **c** the magnified optical microscope image and the corresponding AFM image. **d** the As element mapping of the as-grown b-As_x_P_1-x_ crystal extracted from the intensity of the As 3d XPS peak. **e** A typical photograph of b-As_x_P_1-x_ crystal grown by the conventional growth method. **f** the magnified optical microscope image and the corresponding AFM image. **g** The As element mapping of the as-grown b-As_x_P_1-x_ crystal extracted from the intensity of the As 3d XPS peak.


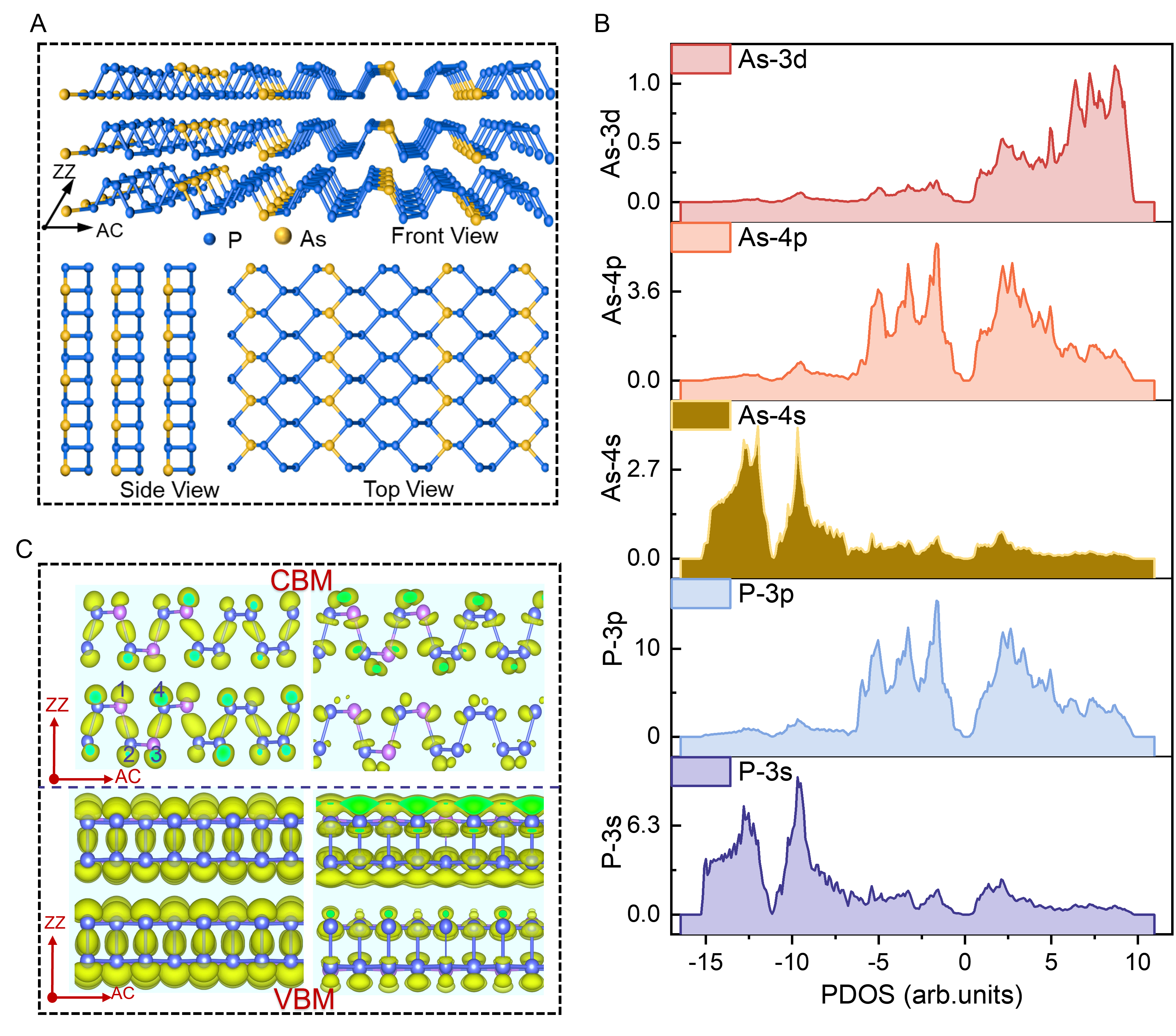


**Fig. S2. First principles calculation.** **a** Crystallographic structure of b-As_0.2_P_0.8_, in both front (upper panel), side, and top (bottom panel) views. **b** The PDOS, and **c** The partial charge density distribution of CBM and VB computed by the DFT method for As_0.2_P_0.8_ crystal.

**Note S2. First principles calculation method.**

Further exploration into the electronic properties of gradually substituting As atoms for P atoms in b-As_0.2_P_0.8_ was conducted through density functional theory (DFT) with the corresponding calculation process. The calculations are computed by using the projector augmented plane-wave method based on the DFT framework in the Vienna ab initio simulation package (VSAP). The generalized gradient approximation of the Perdew-Burke-Ernzerhof function is adopted for electron exchange and correlation (PBE-GGA). A vacuum larger than 20 Å is used to eliminate the interaction between adjacent structures. The cutoff energy for the plane-wave basis set is set to 500 eV, all the structures are fully relaxed with energy and force tolerance of 10^-5^ eV/atom and 10^-3^ eV Å^-1^, respectively. The first Brillouin zone is sampled using a 5×5×1 Monkhorst-Pack grid for relaxation and a 15×15×1 grid for computing the density of states. A supercell structure of 2×3×1 was utilized to facilitate observation of the effects of varying doping concentrations. Additionally, the calculation results of the partial density of states (PDOS) analyses, displayed in Fig. S2b, indicate that the 3p orbital electrons in the b-As_x_P_1-x_ structure exhibit strong contributions near the Fermi level, while the influence of 3s orbital electrons is relatively weak. As atom concentration, the contribution of P-3p orbital electrons decreases, while the effect of As-4p orbital electrons near the Fermi level begins to increase. Figure S2c illustrate the local charge density distribution at the CBM and VBM, where in the VBM, the charge mainly concentrates between 1 and 2, 3 and 4, while in the VBM, the charge predominantly gathers between 1 and 4, 2 and 3, displaying typical covalent bond formations. These results underscore the potential applicability of layered b-As_0.2_P_0.8_ materials in various fields, including electronics and optoelectronics, specifically targeting applications like infrared photodetection, optoelectronic synapses, and optical communications.

**Note S3. Characterization data details of b-As_0.2_P_0.8_ crystal**

Raman spectroscopy was employed to characterize the lattice vibrational modes of b-As_x_P_1-x_ crystal nanosheets as a function of composition with the three characteristic peaks (A_g_^1^, B _2g_, and A _g_^2^). With increasing arsenic content, the intensity of phosphorus-phosphorus (P-P) bond peaks decreases, while the arsenic-arsenic (As-As) bond peaks intensify, consistent with prior studies. Furthermore, a progressive redshift in the Raman peaks correlates with higher arsenic concentrations, providing an efficient and precise means of estimating the crystal composition within b-As_0.2_P_0.8_.

The electronic structure and bonding characteristics of the synthesized samples were probed through X-ray photoelectron spectroscopy (XPS). As shown in Figs. S3a and 3b), the XPS spectra enabled a quantitative compositional analysis of phosphorus and arsenic by integrating the P 3p and As 3d core-level peaks.

X-ray diffraction (XRD) patterns, compared in Fig. S3c, reveal lattice disorder effects due to the distinct lattice parameters of orthorhombic arsenic and phosphorus in b-As_x_P_1-x_. The substitution of phosphorus atoms with larger arsenic atoms within the crystal lattice results in peak broadening and a shift toward lower angles, with the magnitude of this shift increasing alongside arsenic content.

**
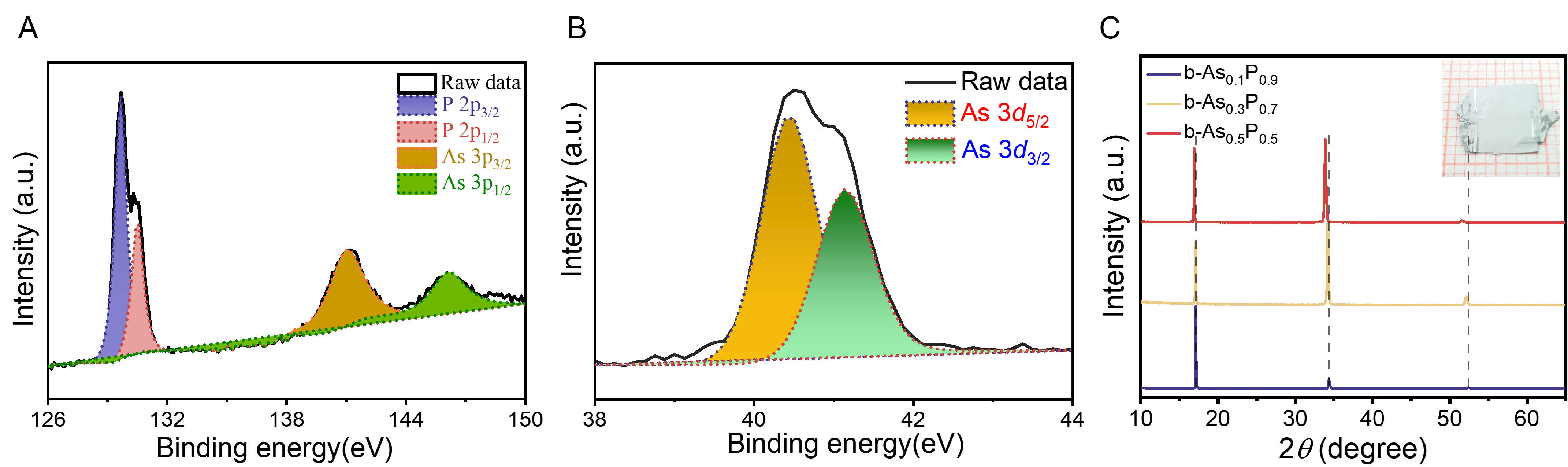
**

**Fig. S3. Characterization of b-As_0.2_P_0.8_ crystal nanosheets. a, b** XPS spectra of P 2p, As 3p, and As 3d core levels on the monolayer b-As_0.2_P_0.8_ crystal nanoplates. **c** XRD spectrum of b-As_0.2_P_0.8_ with different components. The inset shows the optical microscope (OM) image of b-As_0.2_P_0.8_ crystal nanosheets.

**Section S2. Phototransistor Configuration and Polarization-Dependent Performance**


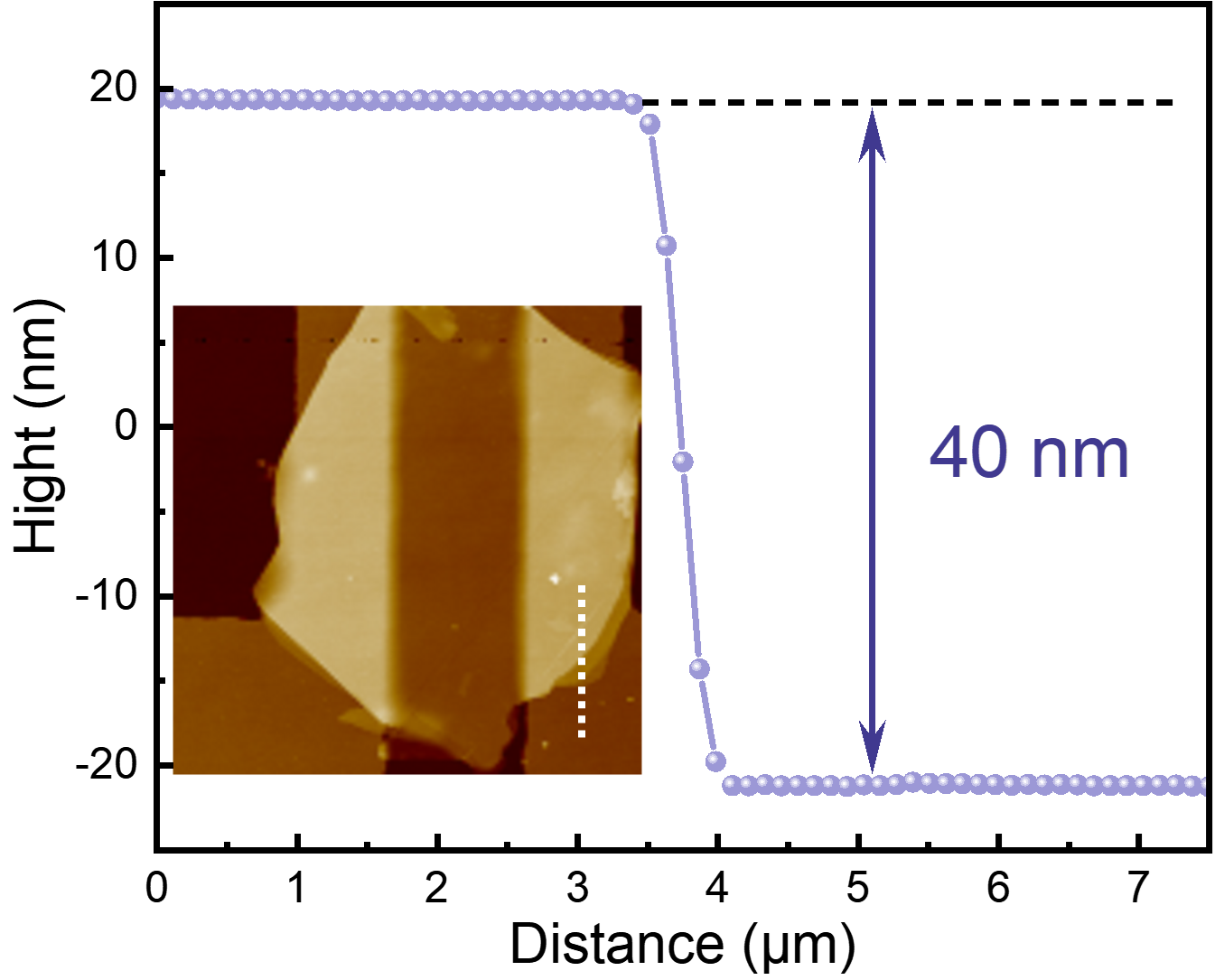


**Fig. S4.** **AFM image of the device.** The inset shows the corresponding thickness map, which is about 40 nm. The exfoliated material displays a uniform thickness, with a corresponding contour plot taken along the electrode-material interface (indicated by the white dashed line) showing a thickness centered around 40 nm.


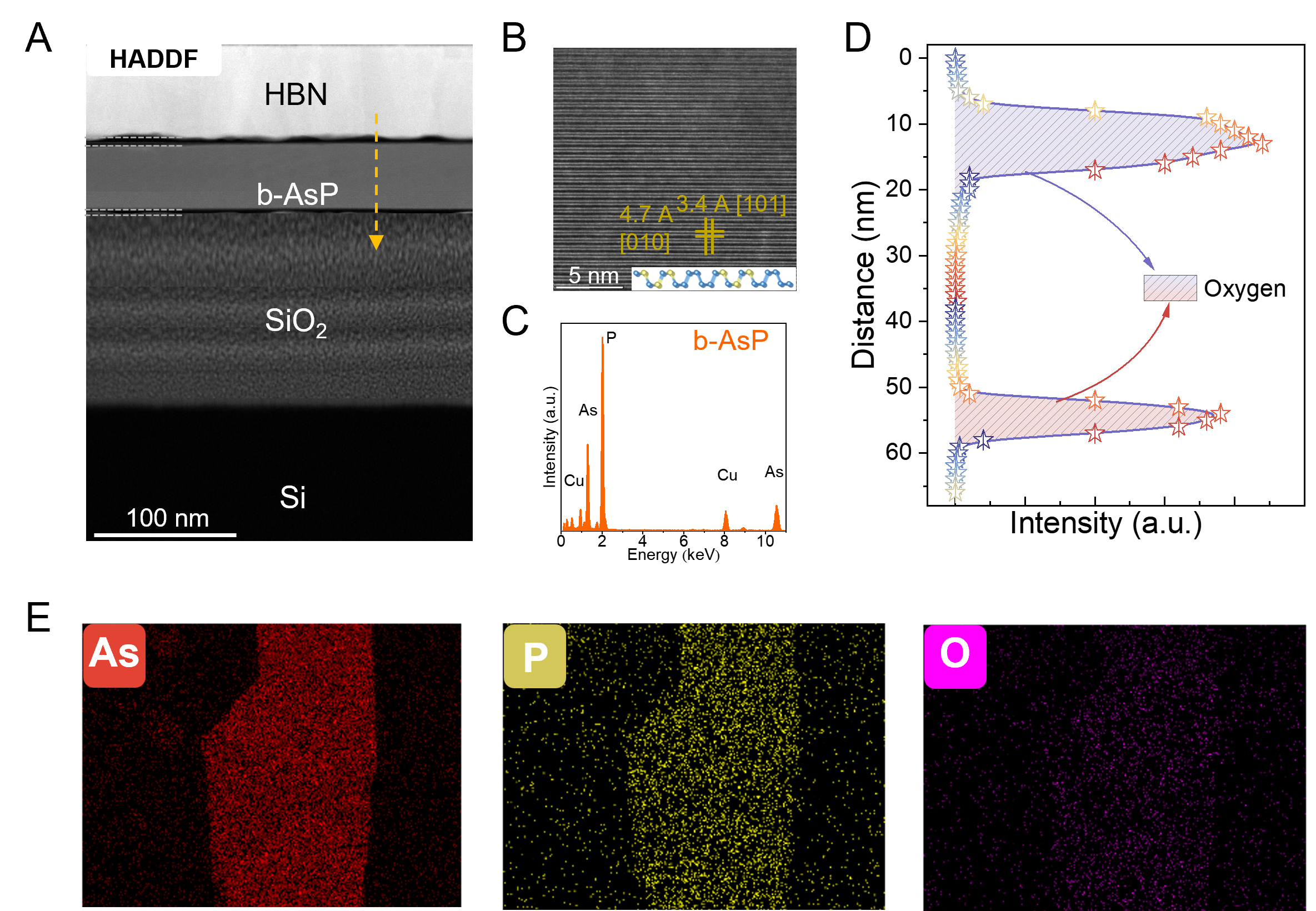


**Fig. S5. Device element analysis and morphology characterization.** **a** Cross-sectional TEM image of the device fabricated on a SiO_2_/Si substrate. **b** The HRTEM images along the interfaces at high magnification. **c** The element intensity distributions. **d** Electron energy loss spectroscopy line scan collected along the vertical dashed line in (a). **e** Corresponding element diagrams of As, P, and O in the SEM image.

**Note S4. Detection mechanism description**

Figure 2c in the main text shows the output characteristic curves (*I*_ds_-*V*_ds_) of the b-As_0.2_P_0.8_ phototransistor under different gate voltages (*V*_g_). During the test, it was found that when a large voltage was applied, the b-As_0.2_P_0.8_ would partially or completely turn black or even ablate and melt. In order to prevent the current passing through the b-As_0.2_P_0.8_ from being too large and causing damage to the material and device, the *V*_ds_ at both ends of the source and drain was controlled between -0.1 V to 0.1 V, and the test range of the *V*_g_ was -60 V to 60 V. It can be seen from the figure that the b-As_0.2_P_0.8_ phototransistor exhibits p-type channel field effect characteristics. When the applied *V*_g_ is small, the holes in the device channel accumulate less and no conductive channel is formed, and the current between the drain and source is small. When a certain *V*_g_ is applied, *I*_ds_ is proportional to *V*_g_ and maintains a good linear relationship, indicating that the contact formed between the channel material b-As_0.2_P_0.8_ and the metal electrode is an ohmic contact. As *V*_g_ increases, the saturation of *I*_ds_ deteriorates, which is mainly due to the low dielectric constant of silicon dioxide. It can also be seen that the slope of the output characteristic curve is different under different *V*_g_, that is, the gate voltage can effectively coordinate the output characteristics of the b-As_0.2_P_0.8_ b-As_0.2_P_0.8_ phototransistor.

When our p-type b-As_0.2_P_0.8_ device is illuminated with 1550 nm light, photons with energy (0.80 eV) greater than the bandgap (0.28 eV) generate electron-hole pairs. The band structure undergoes several key changes: 1)The photogenerated holes enhance the existing majority carrier concentration in the valence band, increasing channel conductivity. 2) Photogenerated electrons (minority carriers) populate the conduction band and, critically, can be captured by trap states located near the conduction band edge. These trap states originate primarily from surface oxidation at the semiconductor-dielectric interface. 3) Under 1550 nm illumination, photogenerated electron-hole pairs form, with holes enhancing channel conduction while electrons can be captured by oxide-related traps. At negative gate voltages, downward band bending accumulates holes in the channel and simultaneously reduces the energy barrier for photogenerated electrons to become trapped at surface defects. These trapped electrons screen hole carriers, causing the observed current depression. At positive gate voltages, upward band bending depletes holes from the channel but also raises the energy level of trap states relative to the conduction band, reducing the barrier for electron release.

The band structure evolution under four key conditions are in Fig. S6:

**Dark state with VG < 0**: Downward band bending at the semiconductor-dielectric interface creates a hole accumulation layer, with the Fermi level positioned close to the valence band edge.

**Dark state with VG > 0**: Upward band bending depletes holes from the interface region, creating a depletion layer with the Fermi level shifting toward mid-gap.

**Light on, VG < 0**: Photogeneration creates electron-hole pairs. Holes enhance channel conductivity while electrons populate trap states. The negative gate field stabilizes trapped electrons by increasing the energetic barrier for de-trapping.

**Light on, VG > 0**: Photogeneration still occurs, but the positive gate field reduces the barrier for electron de-trapping, accelerating their release from trap states.

The channel current of the b-As_0.2_P_0.8_ phototransistor is closely related to the channel length. The shorter the channel length, the smaller the channel resistance and the larger the channel current. According to the transfer and output characteristic curves of the b-As_0.2_P_0.8_ phototransistor, the basic electrical performance parameters of the device are extracted and analyzed. In the phototransistor, the relationship between *I*_ds_ and *V*_ds_ is: *I*_ds_ =Sσ*V*_ds_/L, where L is the length of the conductive channel, S is the cross-sectional area, and σ is the conductivity of b-As_0.2_P_0.8_. When *V*_g_ is zero, the resistance of the device is calculated to be 0.95×l0^4^ Ω. The low resistance value indicates that the quality of the b-As_0.2_P_0.8_ sheet is good and the contact between the metal electrodes at both ends is stable.

The inset in Fig. 2c is the transfer characteristic curve of the back-gate b-As_0.2_P_0.8_ phototransistor. At this time, the *V*_ds_ loaded on the source and drain is 0.1 V, the test range of the *V*_g_ is -60V-60V, and the test temperature is T=300 K. It can be seen from the transfer characteristic curve that when *V*_ds_ is constant, *I*_ds_ decreases with the increase of *V*_g_, and the slope is also gradually decreasing. In the b-As_0.2_P_0.8_ phototransistor, we can use the maximum slope in the *I*_ds_-*V*_g_ curve to obtain the carrier mobility inside the b-As_0.2_P_0.8_ according to the formula. The field effect mobility μ can be expressed by the following formula: μ=$\frac{dI_{ds}}{dV_{g}}\frac{L}{W}\frac{1}{CV_{ds}}$, In the formula, d*I*_ds_/d*V*_g_ is the maximum slope in the transfer curve, that is, the transconductance. *I*_ds_ is the source-drain current; *V*_g_ is the gate voltage; *V*_ds_ is the voltage between the source and the drain, and *V*_ds_=0. 1 V; C is the gate capacitance per unit area, C =ε_0_×ε_r_/d, where ε_0_ is the vacuum dielectric constant, ε_r_ is the relative dielectric constant of SiO_2_; L and W are the length and width of the channel, which are 6 μm and 2 μm, respectively. According to calculations, the hole mobility of the device at room temperature is 381.5 cm^2^V^-1^s^-1^, and the electron mobility is 8.2 cm^2^V^-1^s^-1^.


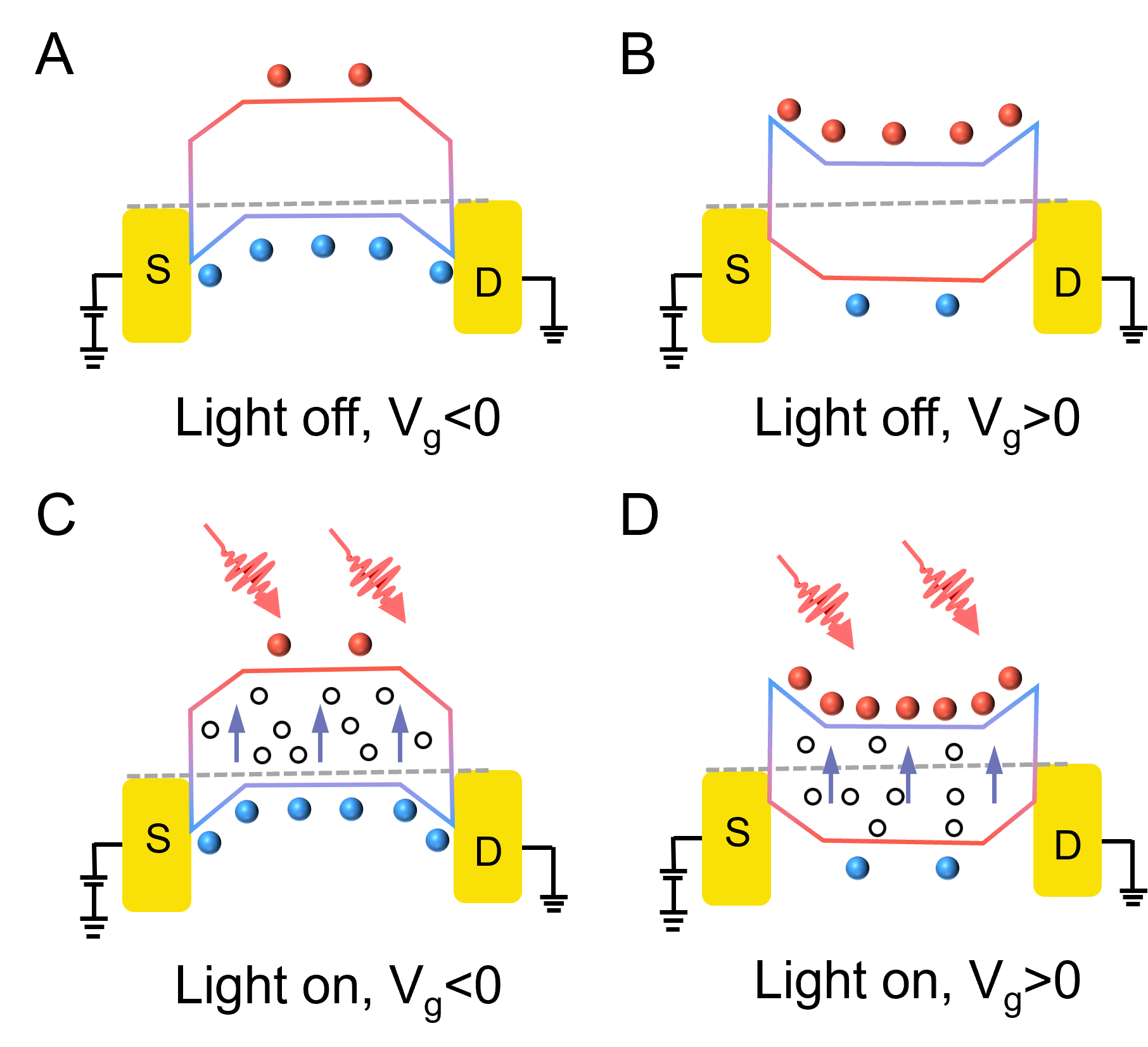


**Fig. S6. The energy band diagrams under different conditions**, such as **a** Light off, *V*_g_<0. **b** Light off, *V*_g_>0. **c** Light on, *V*_g_<0. **d** Light on, *V*_g_>0.

**
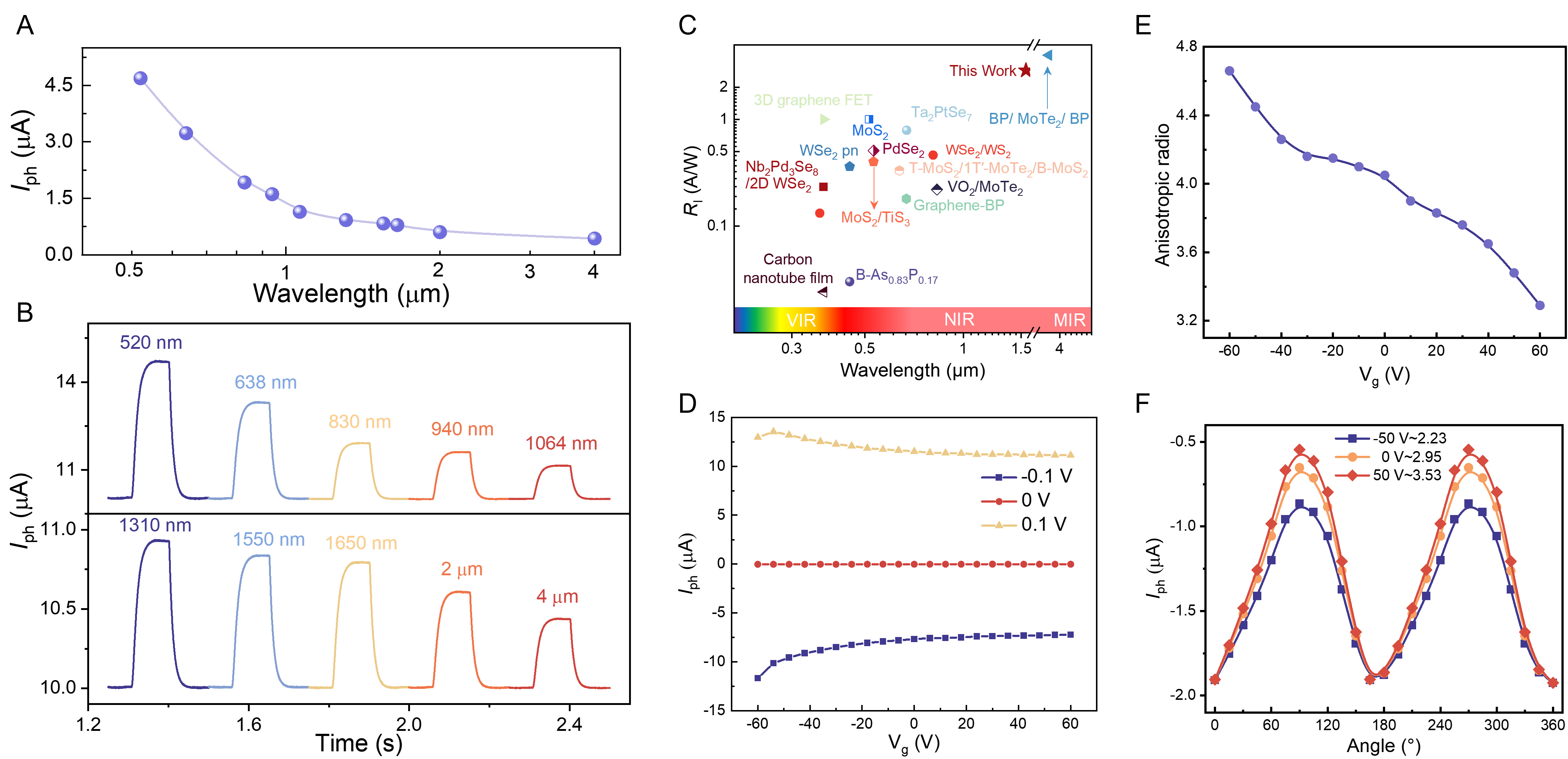
**

**Fig. S7. Photoelectric anisotropy behavior of b-As_0.2_P_0.8_ phototransistors. a** Photocurrent response magnitude of the b-As_0.2_P_0.8_ phototransistor in the 0.52-4 μm range. **b** Corresponding waveform graph of the b-As_0.2_P_0.8_ phototransistor in the 0.52-4 μm range. **c** Comparison of the *R*_I_ of b-As_0.2_P_0.8_ phototransistor with the latest and typical 2D material photodetectors. **d** Transfer characteristic curves of the device under different *V*_ds_ (-0.1 V, 0 V, and 0.1 V). **e** Polarization behavior of the device under different *V*_g_ (-50 V, 0 V, and 50 V). **f** Anisotropy ratio of the device at different *V*_g_ (-60 V to 60 V).

**Table S1. Comparison of the *R*_I_ for different 2D photodetectors.**

| **Materials** | **Wavelength (µm)** | **R_I_ (A W^-1^)** | **Ref.** |
| --- | --- | --- | --- |
| Nb_2_Pd_3_Se_8_/2D WSe_2_ | 0.375 | 0.232 | [1] |
| Re-WSe_2_/WS_2_ | 0.365/0.808 | 0.1319/0.4617 | [2] |
| MoS_2_/TiS_3_ | 0.532 | 0.4 | [3] |
| T-MoS_2_/1T′-MoTe_2_/B-MoS_2_ | 0.638 | 0.331 | [4] |
| Ta_2_PtSe_7_ | 0.671/1.064 | 0.79/0.06 | [5] |
| BP/ MoTe_2_/ BP | 3.7 | 4 | [6] |
| 3D graphene FET | 0.375 | ~ 1 | [7] |
| Graphene-BP | 0.67 | 0.18 | [8] |
| MoS_2_ | 0.516 | 1 | [9] |
| WSe_2_ pn | 0.45 | 0.36 | [10] |
| B-As_0.83_P_0.17_ | 0.45 | 0.03 | [11] |
| VO_2_/MoTe_2_ | 0.83 | 0.22 | [12] |
| Carbon nanotube film | 0.375 | 0.024 | [13] |
| PdSe_2_ | 0.532 | 0.509 | [14] |
| b-As_0.2_P_0.8_ | 0.52-4 | 2.88 | This Work |

**Table S1. Performance Comparison with State-of-the-Art Polarization-Sensitive Neuromorphic Devices.**

| **Material** | **Polarization Ratio** | ***R*_I_ (A W^-1^)** | **Energy/**  **Spike (pJ)** | **PPF** | **Multimodal Capability** | **Ref** |
| --- | --- | --- | --- | --- | --- | --- |
| PbS QDs/Graphene | - | 0.0071 | - | 178% | Polarization +Intensity | [15] |
| Twisted b-AsP homojunctions | 1.1 | 0.15 | - | - | Polarization +Intensity+ Wavelength | [16] |
| Ph-BTBT-10 | 3.85 (365 nm) | 1.47 × 10^4^ | 0.9 × 10^-3^ | - | Polarization | [17] |
| Graphene/MoS_2_ Heterostructure | 1.76 (635 nm) | 105 | - | 115% | Polarization | [18] |
| MoS_2_ | - | - | - | 107.5% | STP+LTP/LTD | [19] |
| h-BN/WSe_2_ heterostructure | - | - | 0.066 | - | LTP/LTD +STDP | [20] |
| MoS_2_/PTCDA | - | - | 10 | 147% | STP+LTP | [21] |
| ZnO/P3HT | - | - | - | 149% | STP+LTP | [22] |
| b-AsP | 4.7  (1550 nm) | 2.88 | 25 | 201% | Polarization +Intensity+ Wavelength +STP+LTP/LTD | This Work |

**Section S3. Neuromorphic Computing Simulation and P****olarization Imaging Application**

**
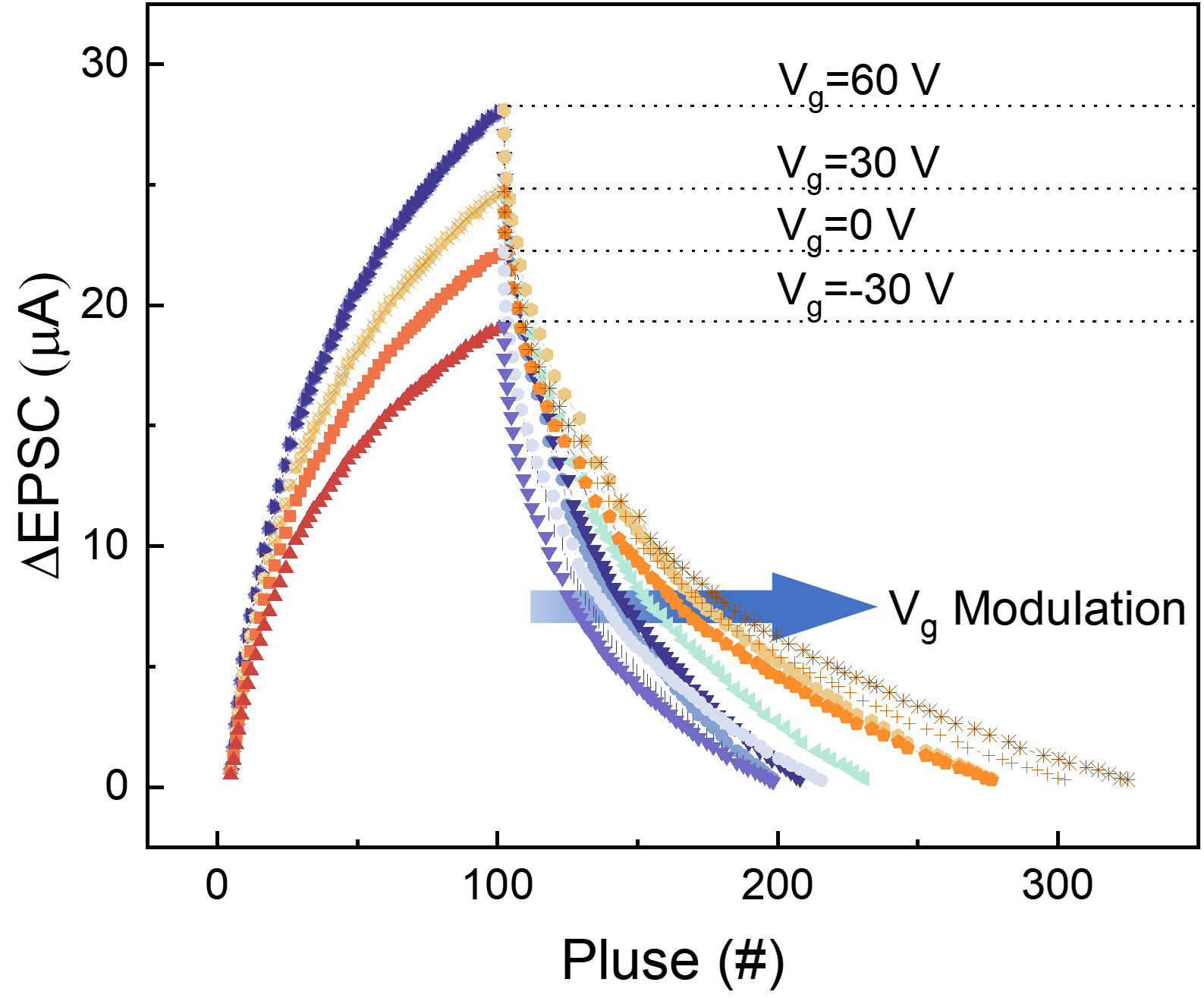
**

**Fig. S8. LTP/LTD curves of the b-As_0.2_P_0.8_ device at different electrical gate voltages.**

**Note S5. Derivation of the relationship between LTP/LTD and time under periodic pulses**

In our b-As_0.2_P_0.8_ optoelectronic memories device, synaptic plasticity, exemplified by long-term potentiation (LTP) and short-term depression (LTD), is modulated via irradiation with near-infrared laser light (1550 nm) at variable polarization states and controlled gate voltages. The weight changes in artificial neural network synapses rely on the LTP/LTD dynamics, tested by adjusting linear polarization angles from 0° to 90°. For defining the weight update rule in our HOENN simulations, the nonlinearity (NL) of the experimentally obtained LTP/LTD curves is calculated across different light pulse widths. The NL values for synaptic potentiation and depression are determined by fitting the LTP/LTD responses as follows:

where, *G*_i_ and *G*_i+1_ are the conductance of *i*^th^ and (*n+1*)^th^ pulses, *G*_min_ and *G*_max_ are the minimum and maximum conductance, *α*_p_ and *α*_d_ are the differences in the conductance between two points on the potentiation and depression curves, *β*_p_ and *β*_d_ are the curvatures of the potentiation and depression curves (i.e., NL), respectively.

For (1-1), by the continuity condition, combined with Taylor expansion, we have:

Comparing (1-1) with (1-3), we have:

Separate the variables and integrate both sides.

We have：

For the LTD process, we also have:

In which, the undetermined coefficients are *α*_p_ and *α*_d_. While *C*_d_ is a constant related to the starting point of timing and does not affect the trend of the curve.


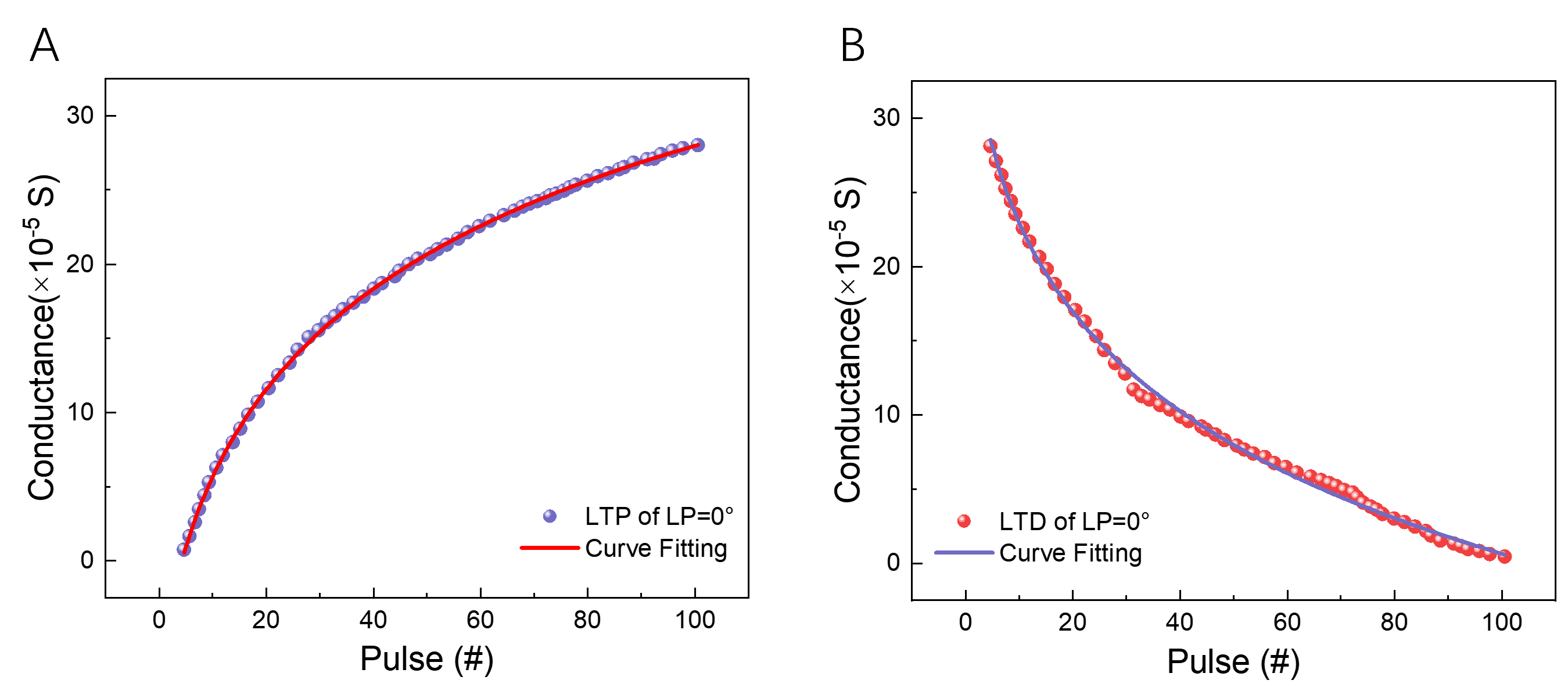


**Fig. S9. a, b The fitting results of the LTP/LTD process under the LP=0° polarization state.**

Specifically, the conductance value, serving as the photoresponse metric in optoelectronic devices, functions equivalently to weight parameters in neural networks. In the simulation architecture, each neural node comprises two complementary devices (*G*^+^_n,m_ and *G*^−^_n,m_), with bidirectional regulation enabling weight updates. For instance, when backpropagation calculations require weight enhancement (Δw>0), the *G*^+^_n,m_ device demonstrates long-term potentiation (LTP) through conductance amplification, while the *G*^−^_n,m_ device exhibits long-term depression (LTD) via conductance attenuation. The equivalent weight of each node is subsequently characterized by the differential value (*G*^+^_n,m_ and *G*^−^_n,m_).

**
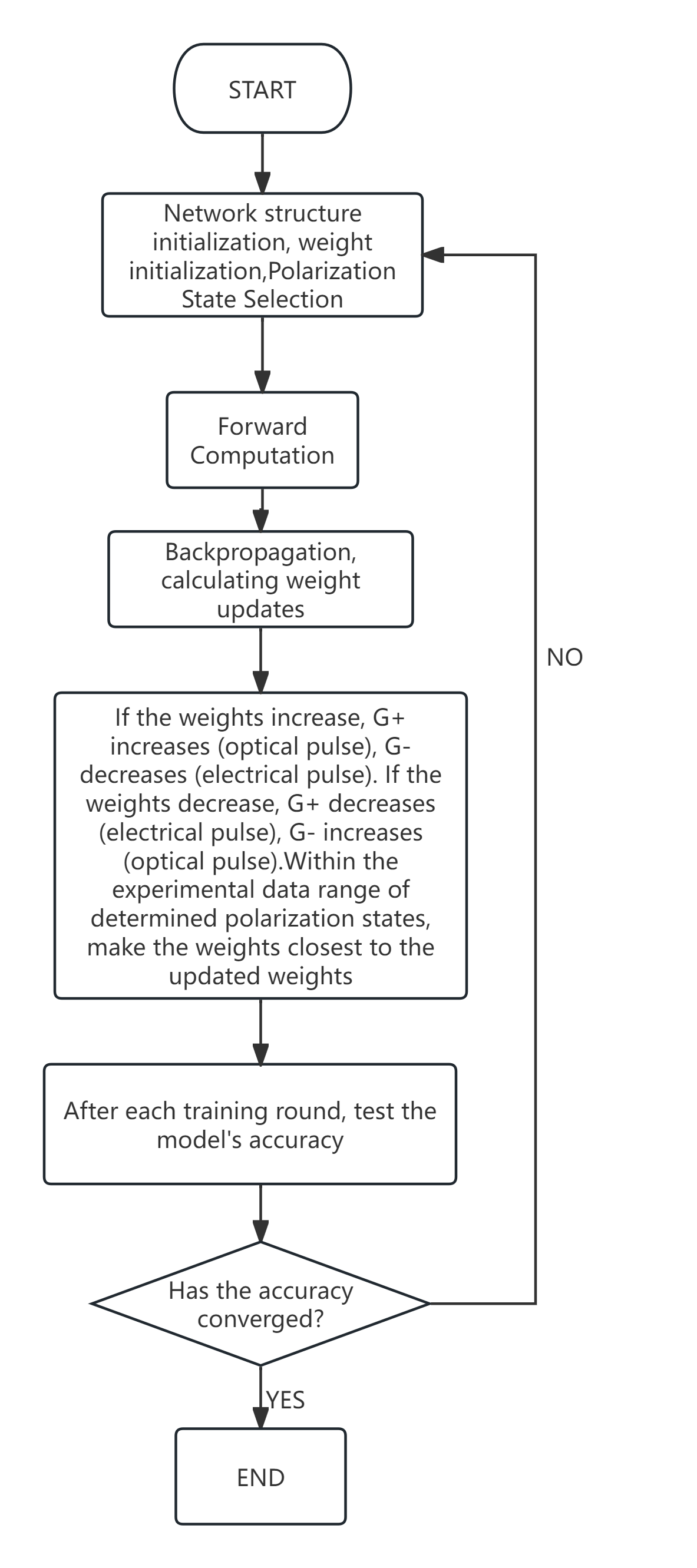
**

**Fig. S10. The training flowchart.**

**Table S2. Extracting LTP/LTD parameters under different polarization attitudes from experimental data.**

| Linear polarization Angle | *G*_min_  (×10^-5^ S) | *G*_min_  (×10^-5^ S) | Fitting parameters LTP | | Fitting  parameters LTD | |
| --- | --- | --- | --- | --- | --- | --- |
|  |  |  | *α*_p_ | *β*_p_ | *α*_d_ | *β*_d_ |
| 0 | 28.00515 | 0.34934 | -3.12E-5 | 11.2 | 8.90E-5 | 13.99 |
| 15 | 27.44783 | 0.34239 | -3.06E-5 | 10.37 | 8.715E-5 | 13.67 |
| 30 | 24.66125 | 0.30763 | -2.73E-5 | 10.15 | 7.83E-5 | 13.14 |
| 45 | 13.93291 | 0.1738 | -1.56E-5 | 9.21 | 4.97E-5 | 12.43 |
| 60 | 13.51492 | 0.16859 | -1.51E-5 | 8.87 | 4.28E-5 | 11.13 |
| 75 | 10.72834 | 0.13383 | -1.19E-5 | 8.46 | 3.37E-5 | 10.79 |
| 90 | 8.49908 | 0.10602 | -1.04E-5 | 8.23 | 2.51E-5 | 10.53 |

**Note S6. Weight Update method**

Simulating Optoelectronic Hybrid Neural Networks Using PyTorch: In this approach, the synaptic weights are represented by the difference in conductance between two devices, that is, *W*=*k* (*G*^+^-*G*^-^), where *k* denotes the amplification coefficient for the current. The changes in the required weights ΔW for each iteration are calculated using the backpropagation algorithm. When Δ*W* >0, *G*^+^_n,m_ increases and *G*^−^_n,m_ decreases, corresponding to the photocurrent pulse, *W* ultimately increases; conversely, when Δ*W* <0, *G*^+^_n,m_, decreases and *G*^−^_n,m_ increases, corresponding to the electric current pulse, *W* ultimately decreases.


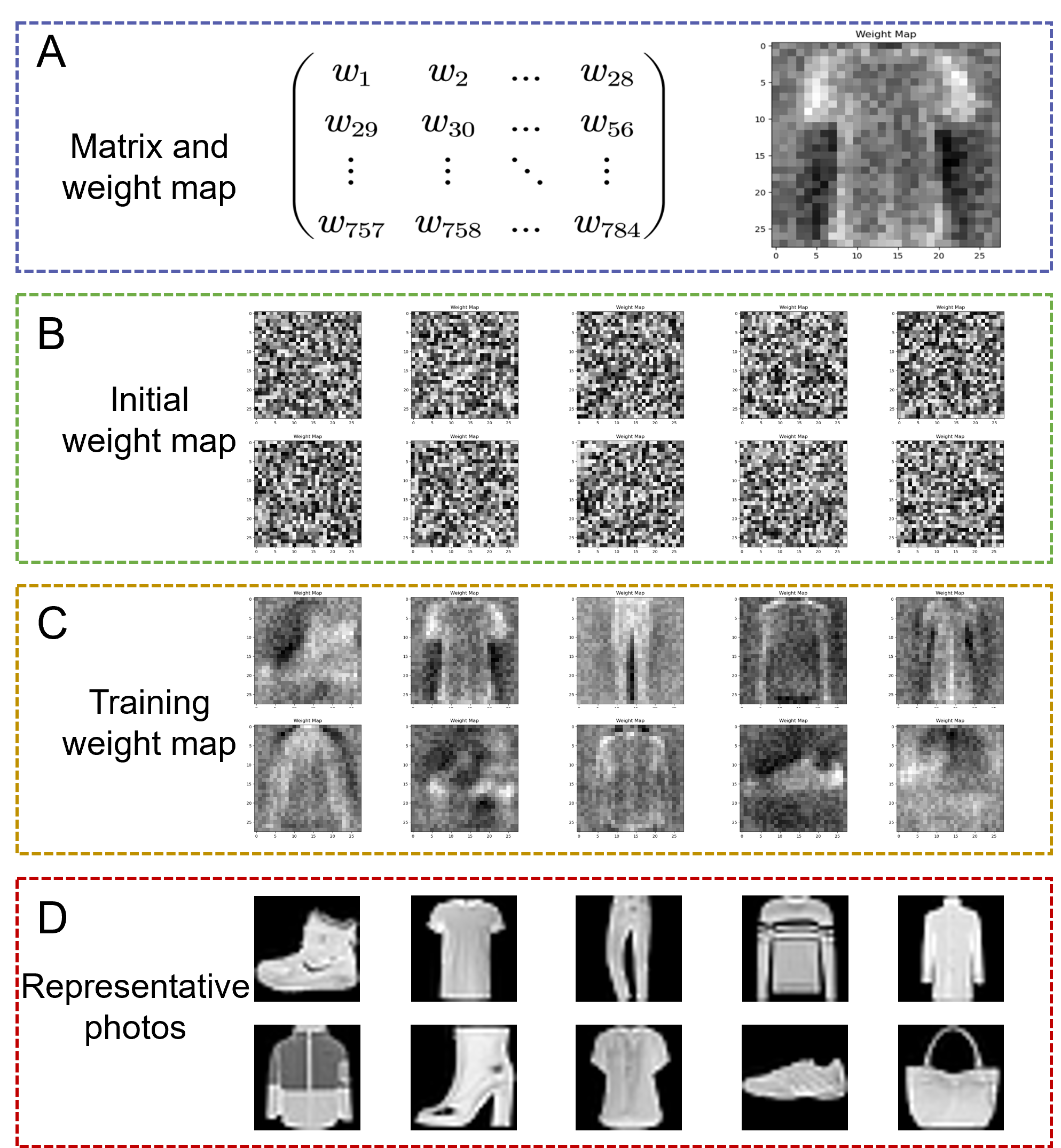


**Fig. S11. Weight map diagram. a** The 784 weights connected to a representative node in the output layer are rearranged into a 28×28 matrix in the classification task, and then the matrix is mapped into the weight map shown in the right panel. **b** The weight mapping connected to the ten nodes in the output layer in the initial state. **c** The weight mapping after training. **d** The typical representatives of each type of image.

**
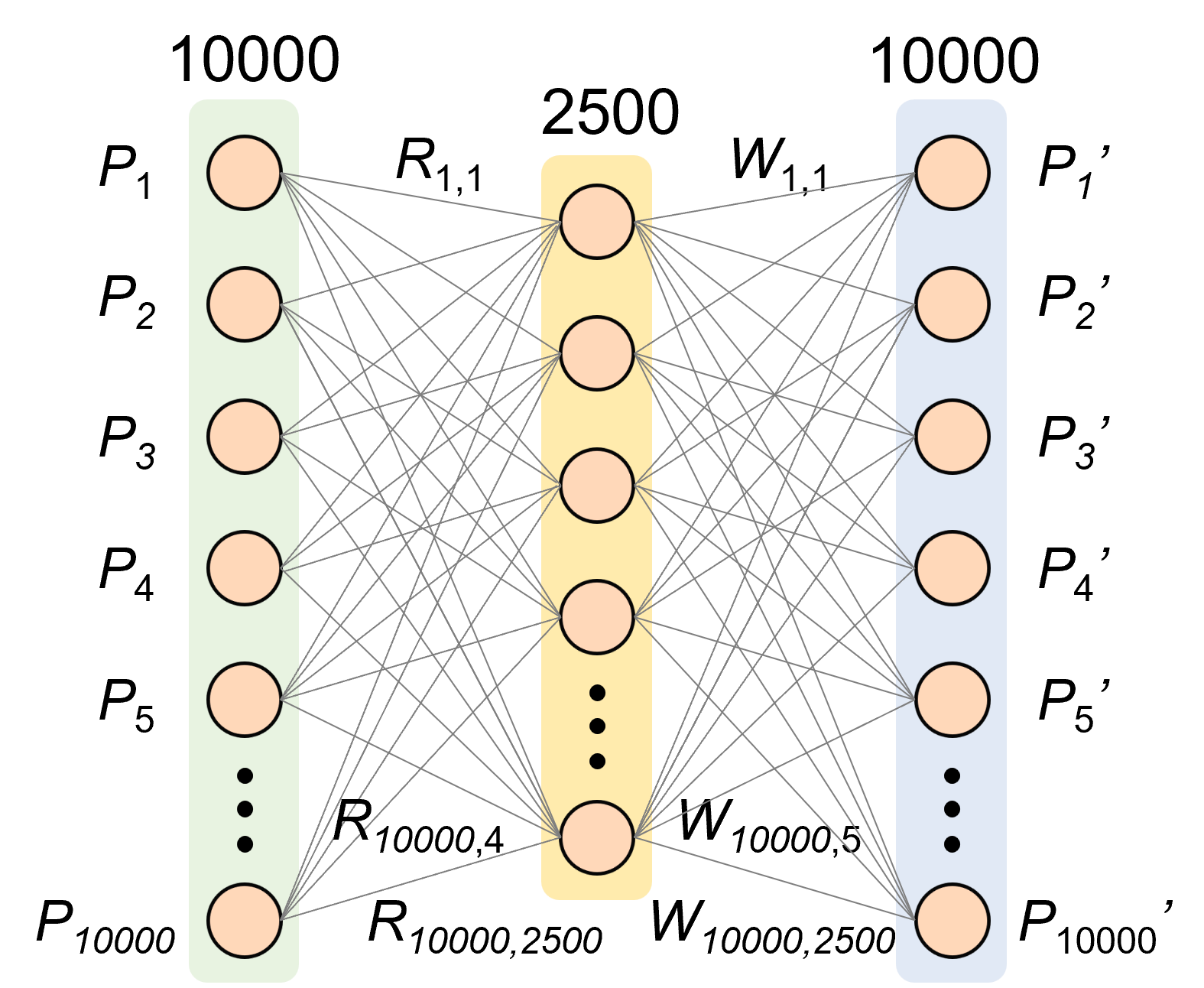
**

**Fig. S12. Schematic diagram of a three-layer encoder-decoder network architecture** of size 10000-2500-10000, aimed at reconstructing images from the Yale Face Database.

**Note S7.** **Reconstruction accuracy calculation method**

The structural similarity is used to calculate the similarity between the reconstructed image and the original image.

The structural similarity is defined as：

Where *μ*_x_, *μ*_y_ is the average value, *σ*_x_, *σ*_y_ is the standard deviation, Z is the covariance of x and y, is a constant used to maintain stability, and *L* is the dynamic range of the pixel. Usually, *k*_1_=0.01, *k*_2_=0.03.

In actual calculation, scikit-image in Python is used to calculate the structural similarity. The example code is as follows:

from skimage.metrics import structural_similarity as ssim

img1 = io.imread (‘img1.png’)

img2 = io.imread (‘img2.png’)

ssim_value = ssim (img1, img2, multichannel=True)

In the reconstruction task, the reconstruction effect of an image is tested after each epoch, and then the structural similarity is calculated.

**Section S4. Multifunctional Stokes polarization imaging**

**
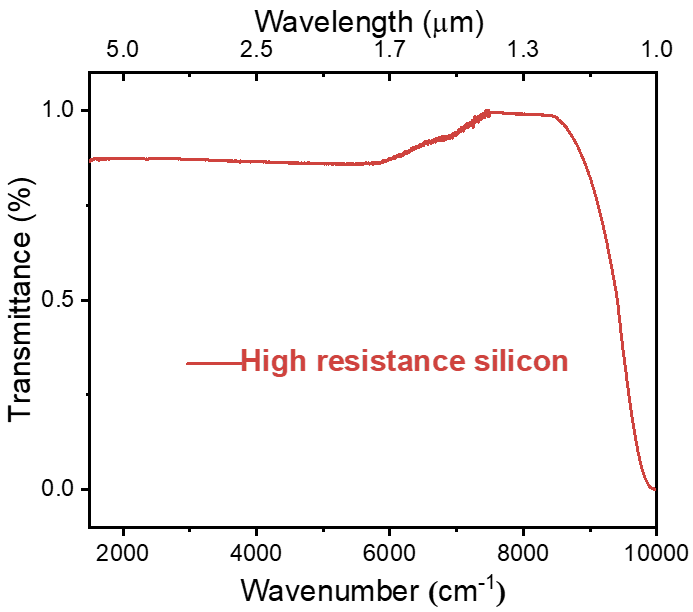
**

**Fig. S13. Infrared transmittance (1 μm to 5 μm) of high-resistance silicon wafer measured by Fourier-transform infrared (FTIR) spectrometer.**

**Note S8. Single-device imaging mechanism**

**a. Scanning-Based Imaging:**

The polarization-resolved images in Figure 5 were acquired using a single-device scanning setup (not a pixelated array). A focused 1550 nm laser spot (diameter≈100 µm) was raster-scanned across a stationary metal mask, while the photocurrents of the b-AsP phototransistor were recorded at each position.

**b. Spatial Resolution:**

The effective resolution is determined by the laser spot size and step size (10 µm). This method is analogous to confocal microscopy and avoids the need for large-scale device arrays. It suggests that the b-AsP based device needs fewer pixels to realize the same resolution for DoLP, making it easier to further promote the imaging resolution.

**Note S9. Polarization state calculation**

The incident polarization information in a polarimeter can be characterized using a set of Stokes vectors. Each component of the Stokes vector is defined as follows:

Where *S*_0_, *S*_1_, *S*_2_, and *S*_3_ represent the four Stokes parameters. *I*_LP-0°_, *I*_LP-45_, *I*_LP-90°_, and *I*_LP-135_ denote the linear polarized intensities measured at angles of 0°, 45°, 90°, and 135°, respectively *I*_R_(x,y) and *I*_L_(x,y) correspond to the right and left circularly polarized intensities, respectively.

Specifically:

$$S_{0}=I_{LP-0^{\circ}}\left( x,y \right)+I_{LP-90^{\circ}}\left( x,y \right)$$

$$S_{1}=I_{LP-0^{\circ}}\left( x,y \right)-I_{LP-90^{\circ}}\left( x,y \right)$$

$$S_{2}=I_{LP-45^{\circ}}\left( x,y \right)-I_{LP-135^{\circ}}\left( x,y \right)$$

Therefore, the image via raster-scanned, the photocurrent response size at each location is obtained. After all the data at polarization angles of 0°, 45°, 90° and 135° are obtained, the image can be reconstructed.

**Stokes Parameter Extraction Workflow:**

**Step 1:** Measure *I*_LP-0°_, *I*_LP-45°_, *I*_LP-90°_, and *I*_LP-135°_at each scan position.

**Step 2:** Compute *S*_0_, *S*_1_, and *S*_2_ using the equations above.

**Step 3:** Normalize parameters to reconstruct polarization-resolved images.

From these parameters, the degree of linear polarization (DoLP) and the angle of linear polarization (AoLP) can be calculated using the following equations:

,

In this work, the circular polarization Stokes parameter (*S*_3_) is set to zero as the device is completely linearly polarized and the *S*_3_ component can be considered to be 0 and not taken into account. Specifically, our device focuses on linear polarization imaging (*S*_0_, *S*_1_, *S*_2_) due to the intrinsic in-plane anisotropy of b-AsP, which inherently responds to linear polarization angles, in this case the *S*_3_ component is 0. Circular polarization (*S*_3_) detection would require additional optical components (e.g., quarter-wave plates) to convert circularly polarized light into linear polarization, which falls outside the scope of this work and it will be our follow-up research work.

**
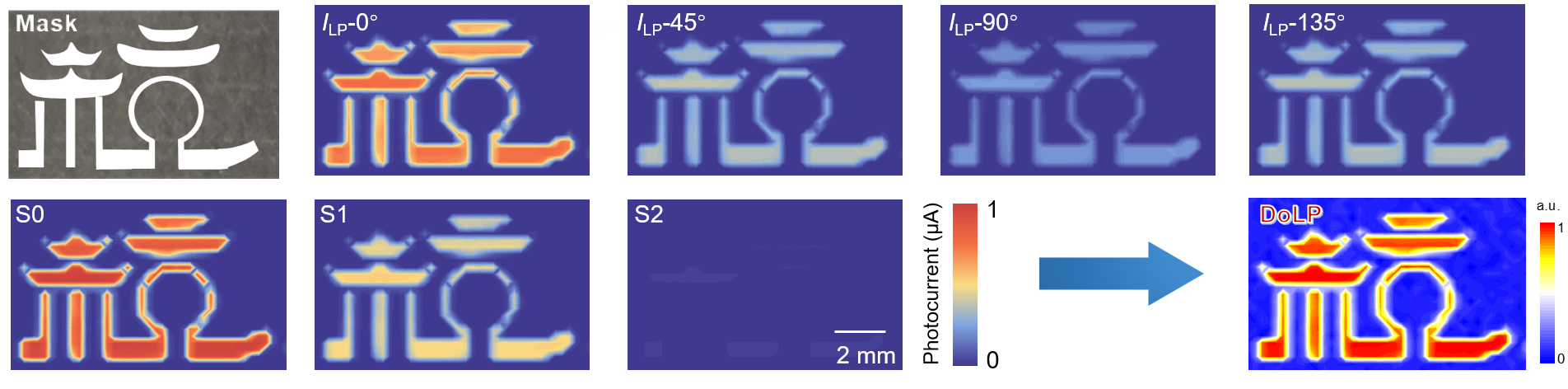
Fig. S14.** **Linear polarization imaging of “hang”** with four different polarization angles 0°, 45°, 90°, and 135° and the DoLP imaging results of the device.

**
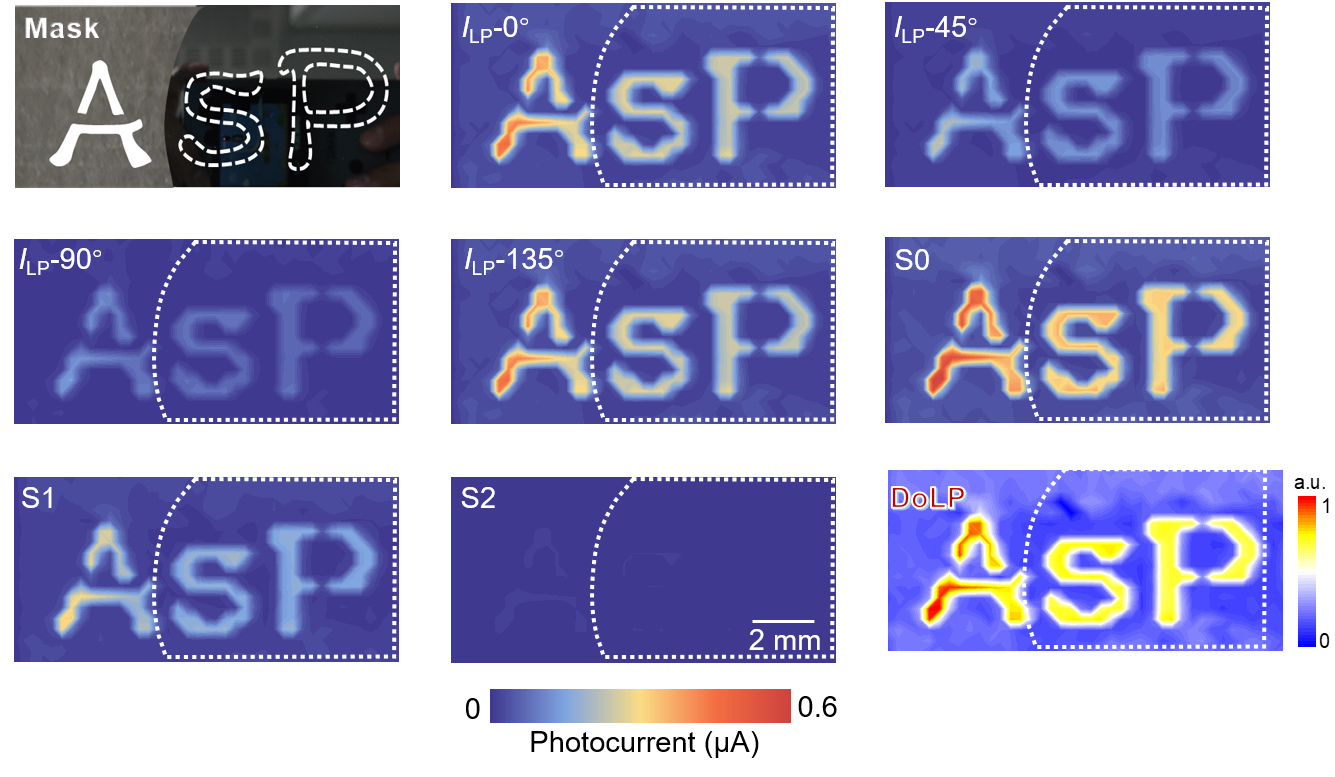
**

**Fig. S15.** **Linear polarization imaging of “AsP”** with four different polarization angles 0°, 45°, 90°, and 135° and the DoLP imaging results of the device.

**Reference**

1. Dat V K, Nguyen M C, Jeong B J, *et al.* Van der Waals Integration of 1D Nb_2_Pd_3_Se_8_ and 2D WSe_2_ for Gate‐Tunable In‐Sensor Image Processing[J]. *Advanced Materials*, 2025: e00011.

2. Yu, L. *et al.* All‐Dielectric Mie Resonances Enabled Giant Anisotropy in High‐Symmetry 2D Material Photodetector: A New Paradigm for Polarized Optoelectronics[J]. *Advanced Functional Materials*, 2025: e17441.

3. Xu, J. et al. Photovoltaic Polarity‐Switching Optoelectronic Device Based on MoS_2_/TiS_3_ Heterostructures for Multifunctional Operation[J]. *Advanced Functional Materials*, e15145.

4. Gao, P. *et al.* High‐Performance Polarization‐Sensitive Photodetectors Based on Fully Depleted T‐MoS_2_/MoTe_2_/B‐MoS_2_ Heterojunction. *Advanced Functional Materials*, (2025): e14834.

5. Yu, H. *et al.* Low‐Symmetry 2D Ta_2_PtSe_7_ Induced by Ultralong Structural Motifs for Flexible Long‐Wave Infrared Photodetection up to 10.6 µm. *Advanced Materials* (2025): e08255.

6. Wang, F. K. *et al.* Bipolar-barrier tunnel heterostructures for high-sensitivity mid-wave infrared photodetection. *Light: Science & Applications* 14.1 (2025): 246.

7. Deng, T. *et al.* Three-dimensional graphene field-effect transistors as high-performance photodetectors. *Nano Letters* 19.3 (2019): 1494-1503.

8. Zhang, X. M. *et al*. High performance mid-wave infrared photodetector based on graphene/black phosphorus heterojunction. *Materials Research Express* 8.3 (2021): 035602.

9. Feng, S. *et al.* A Charge‐Coupled Phototransistor Enabling Synchronous Dynamic and Static Image Detection. *Advanced Materials* (2025): 2417675.

10. Weng, J. L. *et al.* WSe_2_ pn Homojunction Photodetector Engineered by In Situ Ferroelectric Doping. *Advanced Functional Materials* (2025): 2506469.

11.  Long, M. S. *et al.* Room temperature high-detectivity mid-infrared photodetectors based on black arsenic phosphorus. *Science Advanced*, 3.6 (2017): e1700589.

12. Jiang, W. *et al.* A versatile photodetector assisted by photovoltaic and bolometric effects. *Light: Science & Applications* 9.1 (2020): 160.

13. Liu, Y. et al. High-performance, ultra-broadband, ultraviolet to terahertz photodetectors based on suspended carbon nanotube films. *ACS Applied Materials & Interfaces*, 10.42 (2018): 36304-36311.

14. Dai, M. J., *et al.* High-performance, polarization-sensitive, long-wave infrared photodetection via photothermoelectric effect with asymmetric van der Waals contacts. *ACS Nano* 16.1 (2022): 295-305.

15. Lee K J, Kim J H, Jeon S, *et al.* Polarization-Dependent Memory and Erasure in Quantum Dots/Graphene Synaptic Devices[J]. *Nano Letters*, 24.7 (2024): 2421-2427.

16. Wang, F. *et al.* Multidimensional detection enabled by twisted black arsenic–phosphorus homojunctions[J]. *Nature Nanotechnology*, 19.4 (2024): 455-462.

17. Dong, M. *et al.* All‐in‐One 2D Molecular Crystal Optoelectronic Synapse for Polarization‐Sensitive Neuromorphic Visual System[J]. *Advanced Materials*, 36.40 (2024): 2409550.

18. Li, Y. *et al.* Polarization‐sensitive optoelectronic synapse based on 3D graphene/MoS_2_ heterostructure[J]. *Advanced Functional Materials*, 34.15 (2024): 2302288.

19. Wang, B. *et al*. Monolayer MoS_2_ synaptic transistors for high-temperature neuromorphic applications[J]. *Nano Letters*, 21.24 (2021): 10400-10408.

20. Seo S, Jo S H, Kim S, *et al.* Artificial optic-neural synapse for colored and color-mixed pattern recognition[J]. *Nature Communication*, 9.1 (2018): 5106.

21. Wang, S. *et al.* A MoS_2_/PTCDA hybrid heterojunction synapse with efficient photoelectric dual modulation and versatility[J]. *Advanced Materials*, 31.3 (2019): 1806227.

22. Zhao, P. et al. Multifunctional two-terminal optoelectronic synapse based on zinc oxide/poly (3-hexylthiophene) heterojunction for neuromorphic computing[J]. *ACS Applied Polymer Materials*, 4.8 (2022): 5688-5695.
